# WBP2 promotes *BTRC* mRNA stability to drive migration and invasion in triple‐negative breast cancer via NF‐κB activation

**DOI:** 10.1002/1878-0261.13048

**Published:** 2021-08-12

**Authors:** Yvonne Xinyi Lim, Hexian Lin, Tinghine Chu, Yoon Pin Lim

**Affiliations:** ^1^ Integrative Sciences and Engineering Programme National University of Singapore Singapore; ^2^ Department of Biochemistry Yong Loo Lin School of Medicine National University of Singapore Singapore; ^3^ Department of Biomedical Informatics Yong Loo Lin School of Medicine National University Health System Singapore City Singapore; ^4^ National University Cancer Institute Singapore City Singapore

**Keywords:** BTRC, invasion, migration, NFKB, triple‐negative breast cancer, WBP2

## Abstract

WW‐domain‐binding protein 2 (*WBP2*) is an oncogene that drives breast carcinogenesis through regulating Wnt, estrogen receptor (ER), and Hippo signaling. Recent studies have identified neoteric modes of action of WBP2 other than its widely recognized function as a transcriptional coactivator. Here, we identified a previously unexplored role of WBP2 in inflammatory signaling in breast cancer via an integrated proteogenomic analysis of The Cancer Genome Atlas Breast Invasive Carcinoma (TCGA BRCA) dataset. WBP2 was shown to enhance the migration and invasion in triple‐negative breast cancer (TNBC) cells especially under tumor necrosis factor alpha (TNF‐α) stimulation. Molecularly, WBP2 potentiates TNF‐α‐induced nuclear factor kappa B (NF‐κB) transcriptional activity and nuclear localization through aggrandizing ubiquitin‐mediated proteasomal degradation of its upstream inhibitor, NF‐κB inhibitor alpha (NFKBIA; also known as IκBα). We further demonstrate that WBP2 induces mRNA stability of beta‐transducin repeat‐containing E3 ubiquitin protein ligase (*BTRC*), which targets IκBα for ubiquitination and degradation. Disruption of IκBα rescued the impaired migratory and invasive phenotypes in *WBP2*‐silenced cells, while loss of BTRC ameliorated WBP2‐driven migration and invasion. Clinically, the WBP2‐BTRC‐IκBα signaling axis correlates with poorer prognosis in breast cancer patients. Our findings reveal a pivotal mechanism of WBP2 in modulating BTRC‐IκBα‐NF‐κB pathway to promote TNBC aggressiveness.

## Graphical abstract

WW‐domain‐binding protein 2 (WBP2) is a transcriptional coactivator that drives breast cancer progression. Here, we present WBP2 as a neoteric regulator of inflammatory TNF/NF‐κB signaling to promote triple‐negative breast cancer (TNBC) migration and invasion. WBP2 potentiates IκBα ubiquitination by inducing BTRC mRNA stability. Our data reveal new mechanistic insights of WBP2 in modulating posttranscriptional regulation in TNBC.

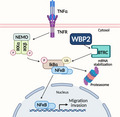

**Abbreviations**
BRCA: breast invasive carcinoma
BTRC: beta‐transducin repeat‐containing E3 ubiquitin protein ligase
DEG: differentially enriched gene
FBXW11: F‐Box/WD repeat‐containing protein 11
IKKα: inhibitor of nuclear factor kappa B kinase alpha
IKKβ: inhibitor of nuclear factor kappa B kinase beta
IκBα: inhibitor of nuclear factor of kappa beta alpha
NFkB: nuclear factor of kappa beta
qPCR: quantitative polymerase chain reaction
TCGA: The Cancer Genome Atlas
TNBC: triple‐negative breast cancer
WBP2: WW‐domain‐binding protein 2

## 1 Introduction

Breast carcinoma is one of the most common cancers among women globally. Despite recent advances in diagnosis and treatment strategies, the mortality for breast cancer remains high [1]. Triple‐negative breast cancer (TNBC), which accounts for about 10–15% of all breast cancers, is particularly notorious for its poor prognosis, lack of effective standard therapies, and high recurrence risk [2]. Chronic inflammation is a hallmark of cancer and a key driver of TNBC metastatic progression [3, 4, 5]. Therefore, an in‐depth understanding of inflammatory responses in TNBC is crucial for facilitating global research efforts to halt TNBC initiation and progression.

The nuclear factor of kappa beta (NFPlease ‐κB) pathway is a key signaling pathway governing inflammatory responses in cancers [6, 7]. The NF‐κB family of transcription factors consist of five members, RelA (p65), c‐Rel, RelB, NF‐κB1 (p50), and NF‐κB2 (p52). These transcription factors are controlled by upstream NF‐κB inhibitor proteins known as IκBs that associate with NF‐κB transcription factors to sequester them in the cytoplasm. Ubiquitin‐mediated degradation is a central mechanism for regulating IκB abundance [8, 9]. IκBα is the most well‐studied member in the IκB family. Upon stimulation of proinflammatory cytokines and chemokines such as TNF‐α, IκBα is phosphorylated by their upstream IKK complex and subsequently recognized by the E3 ubiquitin ligase component, βTrCP [8, 9].

A member of the F‐box protein family, βTrCP, is the substrate recognition component of the SCF^βTrCP^ E3 ubiquitin ligase. It comprises two paralogs, BTRC (also known as βTrCP1) and FBXW11 (also known as βTrCP2 or Hos). Although BTRC and FBXW11 are encoded by different genes, they are structurally and functionally similar [10, 11, 12, 13]. Recognition by βTrCP triggers IκBα ubiquitination and induces subsequent proteasomal degradation of the substrate, hence facilitating the release of NF‐κB dimers into the nucleus [14].

WW‐domain‐binding protein 2 (WBP2) was first identified as a putative ligand of YAP via a functional screen using cDNA library [15, 16]. Immunohistology analysis of clinical breast specimens revealed that WBP2 is upregulated in more than 80% of breast cancer patients, and positively associated with poorer prognosis [17]. Besides breast cancer, WBP2 has been reported to play an oncogenic role in multiple cancer types such as skin, brain, liver, lung, and gastric cancers [18, 19, 20, 21]. The expression and activity of WBP2 is tightly controlled via phosphorylation [22], transcriptional [23], posttranscriptional [18, 24, 25], and post‐translational [17] mechanisms. Mechanistically, WBP2 is progressively revealed to be transcriptional coactivator by binding with ER, YAP, TAZ, and β catenin [17, 18, 22, 26, 27, 28, 29]. However, recent studies implicated WBP2 as an adaptor for LATS2 [21] and a competitive inhibitor for WWC3‐LATS1 complex [20], suggesting that the molecular role of WBP2 may extend beyond transcriptional regulation. To better understand the molecular etiology of WBP2 in breast cancer, we attempt to validate potential molecular mechanisms of WBP2 by performing integrated proteogenomic analysis on TCGA BRCA. We report that WBP2 is involved in the inflammatory pathways in TNBC. We further show that WBP2 is a critical driver of TNF‐α‐induced cell migration and invasion through activating NF‐κB in TNBC. Mechanistically, WBP2 elevates ubiquitin‐mediated proteasomal degradation of IκBα, an inhibitor for NF‐κB, via enhancing mRNA stability of BTRC to promote TNBC cell migration and invasion. In essence, our study suggests a link between WBP2 and inflammation in modulating TNBC migration and invasion and highlights the potential implications of WBP2 as a novel inflammatory regulator.

## 2 Materials and methods

### 2.1 Cell lines and reagents

MCF7, ZR‐75–1, BT549, T47D, HCC1937, MDA‐MB‐231, MDA‐MB‐453, MDA‐MB‐361, SKBR3, MDA‐MB‐468, Hs578t, and MDA‐MB‐436 cells grown as described [17]. All cell lines were obtained from American Type Culture Collection (Manassas, VA, USA). TNF‐α was obtained from GenScript (Piscataway, NJ, USA). Cycloheximide, MG132, Lactacystin, Concanamycin A, and Leupeptin were obtained from Sigma (Burlington, MA, USA). For TNF‐α treatment, cells were first serum‐starved overnight before being treated with 10 ng·mL^−1^ of TNF‐α.

### 2.2 Antibodies

Anti‐WBP2 mouse monoclonal antibody (clone 4CH10) was obtained from EMD Millipore (Billerica, MA, USA). Anti‐GAPH mouse monoclonal antibody was purchased from Pierce (Waltham, MA, USA). Anti‐β tubulin mouse monoclonal and anti‐Flag mouse monoclonal antibodies were purchased from Thermo Fisher Scientific Pierce (Rockford, IL, USA). Anti‐SP1 rabbit polyclonal antibody was purchased from Santa Cruz Biotechnology (Santa Cruz, CA, USA). Anti‐IKKα rabbit, anti‐IKKβ rabbit, anti‐Phospho‐IKKα/β (Ser176/180) rabbit, anti‐IκBα mouse, anti‐Phospho‐IκBα (Ser32/36) mouse, anti‐NF‐κB p65 rabbit, anti‐NF‐κB p65 mouse, and anti‐Phospho‐NFκB p65 (Ser536) rabbit monoclonal antibodies were obtained from Cell Signaling Technology Inc. (Danvers, MA, USA).

### 2.3 Plasmids and siRNA sequences

For plasmids, pcDNA‐v5‐WBP2 and pGEX4T1‐GST‐WBP2 were constructed in our laboratory as previously described [17, 22]. pRL‐TK (Renilla) was purchased from Promega (Madison, WI, USA). pcDNA3 was purchased from Invitrogen (Carlsbad, CA, USA). NF‐κB luciferase reporter plasmid, pHAGE NF‐κB‐TA‐LUC‐UBC‐GFP‐W, was a gift from D. Kotton (Addgene plasmid #4934; Addgene, Watertown, MA, USA). pCMV4‐HA‐IκBα was a gift from W. Greene (Addgene plasmid # 21985). pCMV‐8xHis‐Ub was a gift from W. Kaelin (Addgene plasmid #107392). pcDNA3‐Flag‐BTRC was a gift from P. Howley (Addgene plasmid #10865). For siRNAs, WBP2 siRNAs and luciferase siRNA were performed from Invitrogen. IκBα and BTRC siRNAs were purchased from Integrated DNA Technologies. The siRNA sequences are listed in Table S1.

### 2.4 Transient transfection

Reverse transfection was performed using jetPRIME reagent (Polyplus Transfection, Illkirch, France), according to the manufacturer's instructions.

### 2.5 RNA isolation, reverse transcription, and quantitative polymerase chain reaction

RNA was isolated using PureLink RNA Mini Kit (Ambion, Thermo Fisher Scientific, Austin, TX, USA) and reverse transcribed to cDNA using RevertAid RT Reverse Transcription Kit (Thermo Fisher Scientific). Quantitative real‐time PCR was carried out using ABI TaqMan Fast Universal PCR Master Mix (Thermo Fisher) on Applied Biosystems^®^ 7500 real‐time PCR System (Applied Biosystems, Waltham, MA, USA). Relative gene expression was normalized to housekeeping gene 18S using ΔΔ*C*_t_ method. The list of primers can be found in Table S2.

### 2.6 Protein lysis and immunoblotting

Cells were lysed using ice‐cold nonionic denaturing lysis buffer containing protease and phosphatase inhibitor (Thermo Scientific, Waltham, MA, USA). Equal amounts of proteins were resolved in polyacrylamide gel and transferred to polyvinylidene difluoride fluoride (PDVF) membranes. The membranes were blocked with 1% bovine serum albumin (BSA) (BioWest, Nuaillé, France) and probed with primary antibodies at 4 °C overnight. The next day, the membranes were incubated in horseradish peroxidase (HRP)‐conjugated goat anti rabbit IgG (CST) or goat anti‐mouse IgG (Thermo Scientific). Chemiluminescent signals were detected with Western Bright ECL HRP substrate (Advansta, San Jose, CA, USA) or Amersham ECL Select Western Blotting Detection Reagent (Cytiva, Marlborough, MA, USA) and visualized using ChemiDoc™ Touch Gel Imaging System (Bio‐Rad, Hercules, CA, USA). Image processing and analysis were done using image lab software (Bio‐Rad).

### 2.7 Dual‐luciferase assays

Dual‐Luciferase Reporter Assay System (Promega) was used as per provided protocol. Firefly luciferase and Renila activities were quantified using a Varioskan Lux MultiMode Multiplate reader (Thermo Fisher Scientific). Firefly luciferase signals were normalized to Renilla signals. All assays were run in triplicates.

### 2.8 *In* *vitro* cell‐based assays

For Transwell migration and invasion assays, cells were serum‐starved for 16–24 h. For TNF‐α stimulation, cells are first pretreated with TNF‐α for 8 h. For migration assay, 1 × 10^5^ cells were added in serum‐free media with or without TNF‐α in the top chambers of a 24‐Transwell plate with 8‐μm pore Transwell insert (Corning, New York, NY, USA) and media containing 10% fetal bovine serum was added to the bottom chamber as chemoattractant. For invasion assay, the upper chamber was coated with BD Biosciences Matrigel (BD Biosciences, Bedford, MA, USA); the same number of cells was added in serum‐free media with or without TNF‐α, and media containing 10% fetal bovine serum was used as chemoattractant in the lower chamber. The migration or invasion assay setup was incubated for 16 h at 37 °C. The cells were fixed with 4% paraformaldehyde and cells on the top membrane of the Transwell insert were removed, while the cells on the bottom membrane were stained using crystal violet. Images were acquired using a light microscope at 10× magnification, and 10 random fields of each sample were imaged. Cell viability assay was run in parallel with Transwell assays as a control. Five thousand cells were seeded in triplicate in 96‐well plates. After overnight serum starvation, the cells were treated with TNF‐α for 24 h. The cell viability was measured via The CellTiter 96^®^ Aqueous One Solution Cell Proliferation (Promega) according to the manufacturers' instructions.

### 2.9 Immunofluorescence

Cells were grown on round coverslips. Cells were fixed with 4% paraformaldehyde and permeabilized with 0.2% Triton‐X (Sigma‐Aldrich, Burlington, MA, USA). After blocking with 5% BSA, the cells were incubated with primary antibodies (p65 mouse monoclonal) overnight and then with secondary antibodies conjugated to Alex Fluor (Molecular Probes; Invitrogen, Waltham, MA, USA). The nuclei of the cells were counterstained with Hoechst stain. Coverslips were mounted onto slides with the addition of VECTASHIELD Fluorescent Mounting reagent (Vector Laboratories, Burlingame, CA, USA). The images were acquired using 100× oil immersion objective on a FV3000 confocal laser microscope (Olympus, Shinjuku City, Tokyo, Japan) and analyzed via imagej (National Institute of Health, Bethesda, MD, USA). imagej software was used to determine the nuclear and cytoplasmic intensity of p65. To calculate the nuclear: cytoplasmic ratio, the signal intensity was quantified using fiji (National Institute of Health, Bethesda, MD, USA). The images were split into channels. The blue (Hoechst stain) and red channels (p65 signal) were analyzed. Thresholding was performed to create a binary mask from both images that included all pixels with intensity above background. The Hoechst stain mask was used to generate a nucleus region of interest (ROI) to calculate the nuclear p65 intensity. The cytoplasmic p65 intensity was calculated by subtracting the nuclear p65 intensity from the intensity generated from the p65 stain. Both the nuclear p65 intensity and the cytoplasmic p65 intensity were calculated to generate a nuclear : cytoplasmic ratio.

### 2.10 Subcellular fractionation and *in vivo* ubiquitination assay

Nuclear and cytoplasmic extracts were prepared using the NE‐PER extraction kit (Pierce) as per manufacturer's instructions. *In vivo* ubiquitination assay was performed as previously described in Ref. [30].

### 2.11 Collection and correlation analysis of publicly available datasets

For analysis of correlation between *WBP2* copy number and mRNA expression in TCGA Pan‐cancer Atlas, the copy number alterations, copy number values, and mRNA *z*‐scores (taken from RNA‐Seq v2 SEM and normalized to all samples) were taken from The Cancer Genome Atlas (TCGA) Pan‐cancer Atlas and downloaded from cBioportal (https://www.cbioportal.org/) [31, 32]. WBP2 protein *z*‐scores were generated from Clinical Proteomic Tumor Analysis Consortium (CPTAC) [33] and downloaded from TCGA Firehose Legacy datasets of TCGA BRCA, TCGA OV, and TCGA COADREAD in cBioportal [31, 32]. WBP2 mRNA *z*‐scores were taken from RNA‐Seq v2 SEM platform for TCGA BRCA and TCGA OV but taken from microarray platform for TCGA COADREAD due to the lack of RNA‐Seq data on WBP2 expression in TCGA COADREAD samples. All *z*‐score profiles are generated by cBioportal with all samples as base population. This means that the expression distribution of the gene/protein is estimated by calculating the mean and variance of all samples with expression values. No data transformation and outlier exclusion were performed.

### 2.12 Identification of signaling network associated with WBP2 protein expression in TCGA BRCA

TCGA BRCA samples were segregated into WBP2p_high and WBP2p_low groups according to their median WBP2 protein expression. Differential gene expression analysis between WBP2p_low and WBP2p_high groups was compared using the ‘Group Comparison’ analysis tool provided in cBioportal. A list of differentially enriched genes (DEGs) between the two groups were downloaded from cBioportal and filtered by the following two selection criteria: (a) *P* Value must be < 0.05. (b) |log% FC| ≥ 0.3 or 0.5 (where FC = fold change).

### 2.13 Analyzing TNF signaling components signatures across breast cancer cell lines

The Gene expression‐based Outcome for Breast cancer Online (GOBO) bioinformatic tool (http://co.bmc.lu.se/gobo/) is a bioinformatic tool that enables assessment of microarray‐based gene expression of single or merged gene expression. Normalized gene expression data of 51 breast cancer cell lines were obtained from Neve *et al*. [34]. The gene set for TNF signaling components (named ‘PID_TNF_PATHWAY’) was taken from Schaefer *et al*. [35] and downloaded from the Broad Institute GSA Web site (http://software.broadinstitute.org/gsea/index.jsp) and keyed in as input for in the Gene Set Analysis interface for tumors and cell lines. All genes in the gene list were given equal weight, and the average gene expression was computed prior to stratifying the cancer patients or cell lines based on their gene expression. Categorization of subtypes was performed based on gene expression reported by Hu *et al*. [36] and Parker *et al*. (PAM50) [37].

### 2.14 Correlation of gene expression and survival outcomes in clinical databases

Kaplan–Meier plotter (http://kmplot.com/analysis) was used to assess the effect of genes on survival using breast cancer patients' samples.

### 2.15 Statistical analysis

Data are presented as the mean ± SEM from at least three independent experiments. For all correlation analysis, Spearman's correlation test was performed. For experiments with only two conditions, unpaired *t*‐test was conducted to determine the statistical significance between the two groups. For multiple group comparison, one‐way ANOVA followed by *post hoc* Bonferroni test was performed. A difference was considered significant if *P* < 0.05.

## 3 Results

### 3.1 Analysis of *WBP2* copy number variation, transcript, and protein in TCGA Pan‐cancer Atlas

**Fig. 1:**
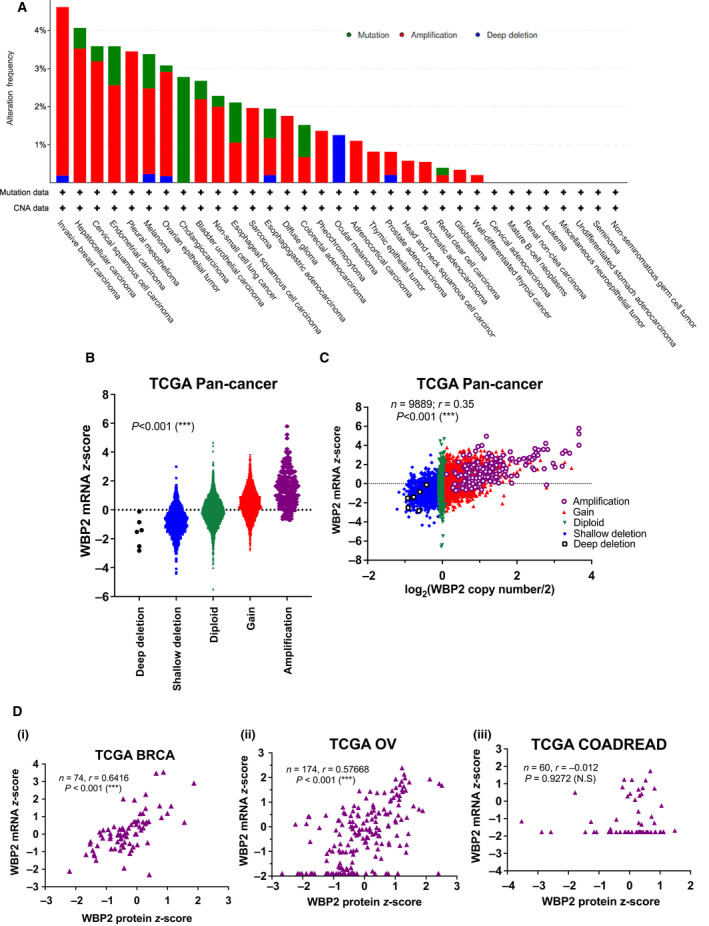
Bioinformatics analysis of *WBP2* in cancers (A) *WBP2* copy number alteration frequency in 32 cancer types from TCGA Pan‐cancer Atlas. The bars are colored according to the type of *WBP2* alterations. The *y*‐axis shows the alteration frequency while *x*‐axis shows each cancer study in The Cancer Genome (TCGA) Pan‐cancer Atlas. (B) Swarm plot of *WBP2* mRNA *z*‐scores in individual samples of TCGA Pan‐cancer Atlas grouped according to *WBP2* copy number alterations. ***P < 0.001(one‐way ANOVA) (C) *WBP2* copy number was transformed on a log2 scale and correlated with *WBP2* mRNA *z*‐scores on a scatter plot. Each dot represents an individual sample that contains matched *WBP2* mRNA *z*‐scores and copy number. The samples are colored according to their *WBP2* copy number alterations. ***P < 0.001 (Spearman's correlation test). (D) Scatter plots indicating Spearman's correlation between *WBP2* mRNA and protein *z*‐scores in (i) TCGA BRCA, (ii) TCGA OV (ovarian serous cystadenocarcinoma), and (iii) TCGA COADREAD (colorectal adenocarcinoma). Each dot represents a sample with matched WBP2 mRNA and protein *z*‐scores. ***P < 0.001; N.S, nonsignificant (Spearman's correlation test).

Although our group has attempted to elucidate the underlying mechanism behind the regulation of WBP2 oncogene, these studies were performed in *in vitro* cell line model or limited clinical samples [17, 22, 23, 25, 26]. There is a lack of comprehensive meta‐analysis of *WBP2* on a genomic, transcriptional, and protein level in large‐scale cancer databases. Since WBP2 has been shown to play oncogenic roles in multiple cancers in the brain, liver, and gastric [18, 19, 21], we wanted to gain a more comprehensive insight into the dysregulation of WBP2 using TCGA Pan‐cancer Atlas, an cancer databases comprising 11 000 samples across multiple tumor types [38]. *WBP2* is observed to be frequently amplified in 25 out of 32 cancer types, and of which, patients with breast invasive carcinoma (BRCA) have the highest frequency of *WBP2* amplification, closely followed by liver and uterine cancers (Fig. 1A).

Copy number amplification is often associated with higher gene expression [39, 40, 41]. The *z*‐score is a transformed and normalized data that reflect the number of standard deviations from the population mean. Therefore, we take *z*‐scores as a representation for gene expression in our analysis. Spearman correlation analysis confirmed that *WBP2* copy number is positively but only moderately correlated with *WBP2* mRNA *z*‐scores across the samples in TCGA Pan‐cancer Atlas (Fig. 1B,C). We noticed a large range of *WBP2* mRNA *z*‐scores for samples with diploid *WBP2* copy number in Fig. 1C, suggesting that *WBP2* mRNA expression is only partially determined by DNA copy number, and likely to be affected by transcriptional or posttranscriptional regulation. Analysis of randomly selected tumor types with high *WBP2* amplification frequency of > 3% [TCGA liver hepatocellular (LIHC), ovarian cancer (OV) and uterine carcinosarcoma (UCS)] and low *WBP2* amplification frequency of < 1% [TCGA colorectal cancer (COADREAD), acute myeloid leukemia (AML), uveal melanoma (UVM)] revealed varying correlation between *WBP2* copy number and mRNA *z*‐scores (Fig. S1). Tumor types with low *WBP2* amplification frequency demonstrated considerably lower correlation coefficients compared to those with high *WBP2* amplification frequency (Fig. S1). This brings about the possibility that in cancer types with lower *WBP2* amplification frequencies, transcriptional or posttranscriptional control of *WBP2* may be the main factor regulating *WBP2* mRNA abundance. On the other hand, *WBP2* mRNA expression seems to be controlled by *WBP2* copy number to a greater extent in TCGA OV and UCS, as compared to the other cancer types.

As WBP2 aberrations can also occur on the protein level [17, 23, 26, 42], we analyzed the relationship between WBP2 mRNA and protein expression in individual patients of the following three cancer types, BRCA, ovarian cancer (OV), and colorectal cancer (COADREAD). These three cancer types were selected due to their availability of mass spectrometry data from Clinical Proteomic Tumor Analysis Consortium (CPTAC). Samples without corresponding vales for *WBP2* mRNA or protein *z*‐scores in each tumor types were not considered in the analysis. Our results demonstrated that *WBP2* mRNA is moderately and positively associated with protein expression in TCGA BRCA and OV, with a Spearman correlation of around 0.6. However, there is no significant correlation in TCGA COADREAD (Fig. 1Di–iii). These data suggest that (a) *WBP2* is likely to be differentially regulated in various cancer types and (b) *WBP2* level is likely to be determined not only by transcriptional or posttranscriptional mechanism, but also at the translational and post‐translational levels. Therefore, WBP2 protein expression is likely to a better reference than gene expression or copy number to evaluate the effects of WBP2 in cancer progression.

### 3.2 WBP2 is associated with inflammatory pathways in breast cancer

**Fig. 2:**
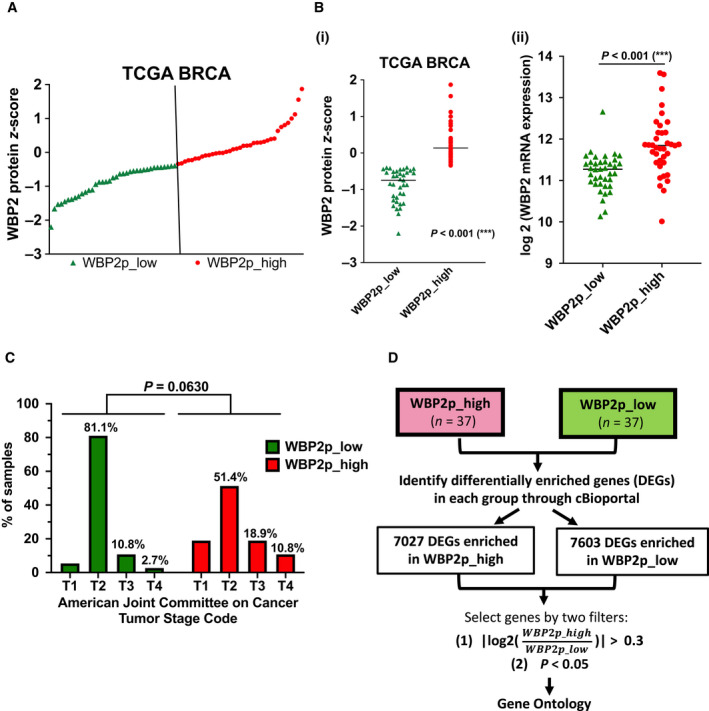
Higher WBP2 protein expression is associated with higher *WBP2* mRNA and copy number alterations, as well as breast cancer progression in TCGA BRCA (A) TCGA BRCA samples were arranged according to WBP2 protein *z*‐scores and stratified into two groups according to their median (vertical lines). The two groups are termed as ‘WBP2p_low’ (green triangle) and ‘WBP2p_high’ (red dot). (B) Comparison of (i) WBP2 protein *z*‐scores and (ii) log_2_(*WBP2* mRNA expression) ***P < 0.001 (unpaired *t*‐test) (C) Percentage of samples in each stage of American Joint Committee on Cancer (AJCC) stage code was computed and plotted on a bar char. Chi‐squared test was performed to determine the statistical significance. (D) Workflow to filter and select the DEGs in WBP2p_high and WBP2p_low groups. The significance of DEGs in the two groups was derived from Student's *t*‐test.

Since WBP2 has the highest amplification frequency in breast cancer, we proceeded to study the effects of WBP2 in breast cancer. Given that WBP2 upregulation was observed at higher breast cancer stages via immunohistochemistry analysis [17], we speculated that WBP2 protein expression in TCGA BRCA samples can be similarly associated with breast cancer stage progression. To achieve this, TCGA BRCA patients with available mass spectrometry data were selected and stratified into two groups based on their median WBP2 protein expression (referred to as ‘WBP2p_high’ and ‘WBP2p_low’) (Fig. 2A and Table S3). Samples in WBP2p_high group were demonstrated to have higher WBP2 protein and mRNA levels (Fig. 2Bi‐ii), thereby confirming our findings in Fig. 1. Interestingly, clinical analysis revealed that higher WBP2 protein expression correlates with stage progression in TCGA BRCA (Fig. 2C). However, the difference between the two groups was *P* > 0.05, and this could be attributed to the small sample size (*n* = 74) of TCGA BRCA samples with available mass spectrometry data on WBP2 protein abundance.

To delineate potential mechanisms regulated by WBP2, we analyzed the signaling network associated with WBP2 protein expression in TCGA BRCA. Since transcriptional regulation is a primary consequence of a myriad of signaling pathway activation [43], we analyzed the DEGs between WBP2p_low and WBP2p_high groups will reflect the signaling network associated with WBP2 protein levels. The DEGs in both WBP2p_low and WBP2p_high groups were selected based on the following two filters: (a) *P* ≤ 0.05 and (b) |log_2_FC| ≥ 0.3 [i.e., fold change (FC) > 1.23] (Fig. 2D and Table S4). The fold change in 1.23 is well within the benchmark that is widely adopted in transcriptomic studies [26, 44, 45]. Enrichment analysis of selected DEGs revealed that WBP2 is involved in multiple signaling pathways (Table 1). Several pathways such as Wnt [17, 22, 26] and EGFR [39] had been previously reported to be associated with WBP2, thereby increasing the validity of this integrated proteogenomic study. Additionally, other novel pathways were also revealed to be linked to WBP2, such as cadherin signaling, gonadotropin‐releasing hormone receptor, and integrin and p53 pathways. Interestingly, gene ontology analysis showed WBP2 protein expression to be associated with DEGs involved in inflammatory pathways such as chemokine and cytokine signaling, TGFβ signaling, and Toll receptor pathway (Table 1). This finding coincides with our previous RNA‐Seq analysis in which WBP2 was implicated in various inflammatory response pathways such as TNF signaling, cytokine–cytokine receptor interaction, NF‐κB pathway, and Toll‐like signaling [26].

**Table 1:** Gene Ontology (GO) analysis of DEGs in both WBP2p_high and WBP2p_low groups using PANTHER database (*P* < 0.05; |log2FC| > 0.3; inflammatory signaling highlighted in red).

PANTHER pathways	Gene count
Wnt signaling pathway (P00057)	16
Cadherin signaling pathway (P00012)	12
Gonadotropin‐releasing hormone receptor pathway (P06664)	11
Alzheimer disease‐presenilin pathway (P00004)	9
Angiogenesis (P00005)	8
Heterotrimeric G‐protein signaling pathway‐Gi alpha and Gs alpha‐mediated pathway (P00026)	7
Metabotropic glutamate receptor group III pathway (P00039)	6
EGF receptor signaling pathway (P00018)	6
p53 pathway (P00059)	5
Ionotropic glutamate receptor pathway (P00037)	5
Inflammation mediated by chemokine and cytokine signaling pathway (P00031)	5
FGF signaling pathway (P00021)	5
Alzheimer disease‐amyloid secretase pathway (P00003)	4
Adrenaline and noradrenaline biosynthesis (P00001)	4
TGF‐beta signaling pathway (P00052)	4
Huntington disease (P00029)	4
VEGF signaling pathway (P00056)	3
Transcription regulation by bZIP transcription factor (P00055)	3
Toll receptor signaling pathway (P00054)	3
Parkinson disease (P00049)	3
PDGF signaling pathway (P00047)	3
Muscarinic acetylcholine receptor 2 and 4 signaling pathway (P00043)	3
Metabotropic glutamate receptor group I pathway (P00041)	3
Integrin signaling pathway (P00034)	3
p53 pathway feedback loops 2 (P04398)	3
Ras pathway (P04393)	3
Heterotrimeric G‐protein signaling pathway‐Gq alpha and Go alpha‐mediated pathway (P00027)	3
Dopamine receptor‐mediated signaling pathway (P05912)	3
B‐cell activation (P00010)	3

### 3.3 WBP2 promotes TNF‐α‐induced cell migration and invasion in TNBC

**Fig. 3:**
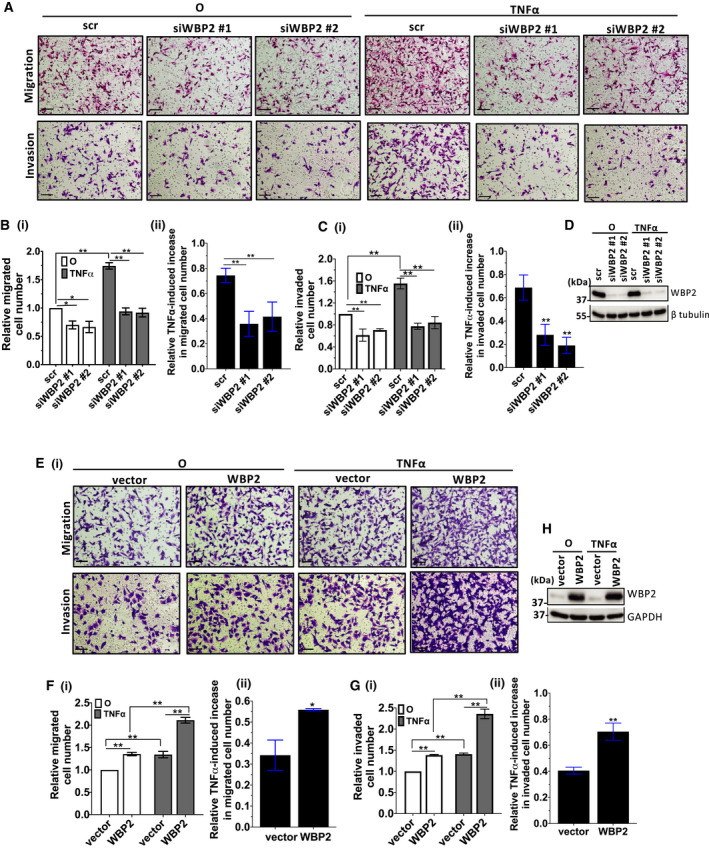
WBP2 positively regulates TNF‐α‐induced cell migration and invasion and TNF target genes (A–D) Cell migration and invasion assays in WBP2‐silenced MDA‐MB‐231 cells. (A) Representative images from Transwell migration and invasion assays of MDA‐MB‐231 cells. Cells from 10 random fields of each sample were imaged. Magnification: 10×, scale bar (black line on bottom left): 250 µm. (B) (i) Relative number of migrated cells was quantified and normalized to unstimulated control (O). (ii) Fold increase in migrated cells by tumor necrosis factor alpha (TNF‐α) was calculated using the formula: ((TNF‐α‐stimulated migrated cell number‐nonstimulated cell number(O))/nonstimulated migrated cell number(O)) for each sample. (C) (i) Relative number of invaded cells was quantified and normalized to unstimulated control (O). (ii) Relative fold increase in invaded cells induced by TNF‐α was determined by the formula: ((TNF‐α‐stimulated invaded cell number‐nonstimulated invaded cell number(O))/nonstimulated invaded cell number(O)) for each sample. (D) Western blot was performed to confirm WBP2 knockdown. (E–G) Cell migration and invasion assays in WBP2‐overexpressed BT549 cells. (E) Representative images of Transwell migration and invasion assays in BT549 cells. Cells from 10 random fields of each sample were imaged. Magnification: 10×, scale bar (black line on bottom left): 250 µm. (F) (i) Relative number of migrated cells was quantified and normalized to unstimulated control (O). (ii) Fold increase in migrated cells by TNF‐α was calculated using the formula: ((TNF‐α‐stimulated migrated cell number‐nonstimulated cell number(O))/nonstimulated migrated cell number(O)) for each sample. (G) (i) Relative number of invaded cells was quantified and normalized to unstimulated control (O). (ii) Relative fold increase in invaded cells induced by TNF‐α was determined by the formula: ((TNF‐α‐stimulated invaded cell number‐nonstimulated invaded cell number(O))/nonstimulated invaded cell number(O)) for each sample. (H) Western blot to confirm WBP2 overexpression. For all experiments in this figure, error bars annotate SEM, *n* = 3, *P < 0.05, **P < 0.01 (unpaired *t*‐test for two group comparison; one‐way ANOVA followed by *post hoc* Bonferroni test for multiple group *t*‐test).

TNF‐α is a proinflammatory cytokine that is upregulated in breast cancer and correlates with breast cancer progression and recurrence [46, 47, 48, 49, 50]. According to our previous RNA‐Seq analysis in MDA‐MB‐231 TNBC cell line, TNF signaling appears to be a top‐hit inflammatory pathway potentially regulated by WBP2 [26]. Since merged expression of TNF signaling components was shown to be the most enriched in TNBC breast cancer cell lines (Fig. S2), TNBC was used as the cell model for this Migration and invasion are two key phenotypes that are induced by TNF‐α and linked to cancer recurrence [51, 52, 53, 54, 55, 56]. Furthermore, the phenotypic role of WBP2 in migration and invasion has not been as extensively studied in TNBC cell models as compared to other phenotypes such as growth by our lab and others [17, 23, 25, 26, 42]. Therefore, we investigated the effects of WBP2 in TNF‐α‐stimulated migration and invasion. WBP2 was silenced in MDA‐MB‐231, a TNBC cell line known to have high WBP2 expression, using two different WBP2‐targeting siRNAs. Reduced WBP2 expression was observed to slightly diminish cell migration and invasion at basal conditions, but drastically impaired TNF‐α‐induced migration and invasion (Fig. 3A–D). Consistently, overexpression of WBP2 in low WBP2‐expressing BT549 cells markedly enhanced cell migration and invasion at both basal and TNF‐α conditions (Fig. 3E–H). The fold increase in cell migration and invasion induced by TNF‐α was also significantly enhanced with WBP2 overexpression (Fig. 3Fii,Gii). To further confirm the modulation of WBP2 on TNF signaling, the effects of WBP2 on selected TNF‐α‐induced genes were analyzed by qPCR. WBP2 markedly inhibits the expression of all target genes upon TNF‐α stimulation (Fig. S3). In a nutshell, WBP2 is likely to be important for TNF‐α‐induced migration and invasion processes, as well as regulation of TNF‐α‐induced target genes in TNBC.

### 3.4 WBP2 activates TNF‐α‐induced NF‐κB activity via limiting IκBα protein abundance in TNBC

**Fig. 4:**
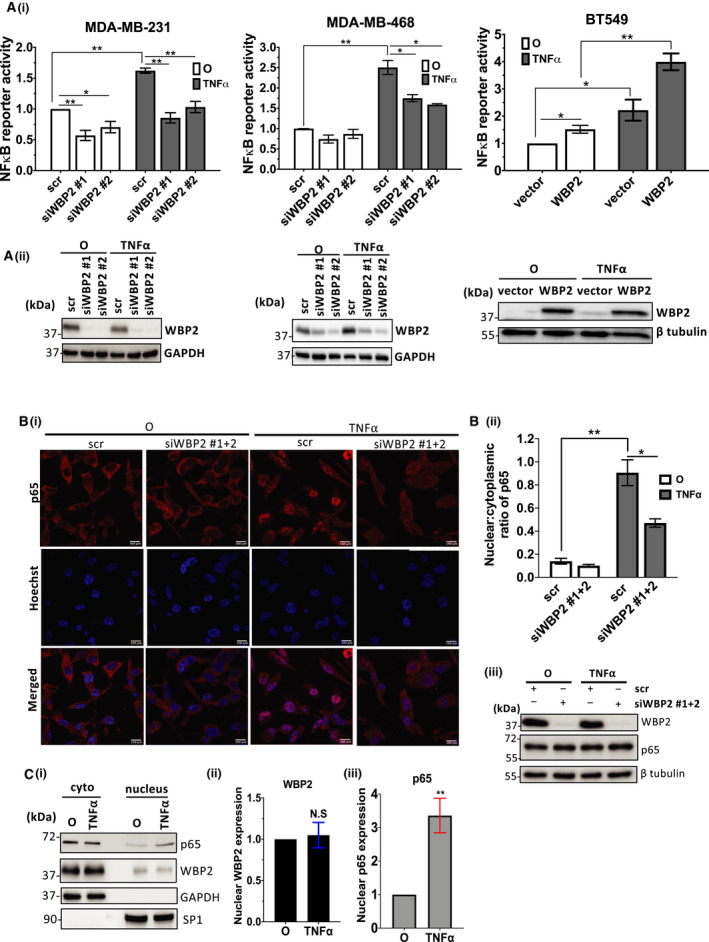
WBP2 activates TNF‐α‐induced NF‐κB activity in TNBC cells. (A) WBP2 positively regulates tumor necrosis factor alpha (TNF‐α)‐induced nuclear factor of kappa beta (NF‐κB) transcriptional activity in MDA‐MB‐231, MDA‐MB‐468, BT549 (i) NF‐κB reporter assay performed in three TNBC cell lines. Cells were harvested after 6h of TNF‐α stimulation. Luciferase signals representing NF‐κB reporter activity were normalized with promoter Renilla signals and quantified related to scrambled (scr) siRNA/vector control cells unstimulated with TNF‐α. (ii) Immunoblot to validate WBP2 knockdown or overexpression. The data are represented as the mean ± SEM of three independent experiments. *P < 0.05, **P < 0.01 (one‐way ANOVA followed by *post hoc* Bonferroni test). (B) WBP2 silencing reduced TNF‐α‐stimulated nuclear accumulation of NF‐κB p65 subunit in MDA‐MB‐231 cells. (i) Representative immunofluorescence (IF) images showing p65 (red) localization upon knockdown of WBP2 and 30min of TNF‐α stimulation in MDA‐MB‐231 cells. O represents cells that are not treated with TNF‐α. Pooled WBP2 siRNAs (siWBP2 #1 + 2) were generated by combining siWBP2 #1 and siWBP2 #2 in equal amount. Nuclei were stained with Hoechst (blue). Scale bar (white; bottom right), 100µm. White arrow indicates examples of cells with p65 localized in the nucleus. (ii) The nuclear:cytoplasmic ratio of p65 was quantified using imagej software. The data are represented as the mean ± SEM, *n* = 3. *P < 0.05, **P < 0.01 (one‐way ANOVA followed by *post hoc* Bonferroni test). (iii) Immunoblot to confirm efficiency of WBP2 knockdown and determine total cellular p65 expression. β‐Tubulin was used as the loading control. (C) (i) Representative immunoblot of subcellular fractionation experiment showing WBP2 and p65 nuclear and cytoplasmic expression in MDA‐MB‐231 cells after 30 min of TNF‐α induction. (ii&iii) Densitometry analysis of (ii) nuclear WBP2 and (iii) p65 signal was performed to confirm the nuclear expression after TNF‐α stimulation. The data are represented as the mean ± SEM, *n* = 3, *n* = 3. N.S, nonsignificant, **P < 0.01 (unpaired *t*‐test).

**Fig. 5:**
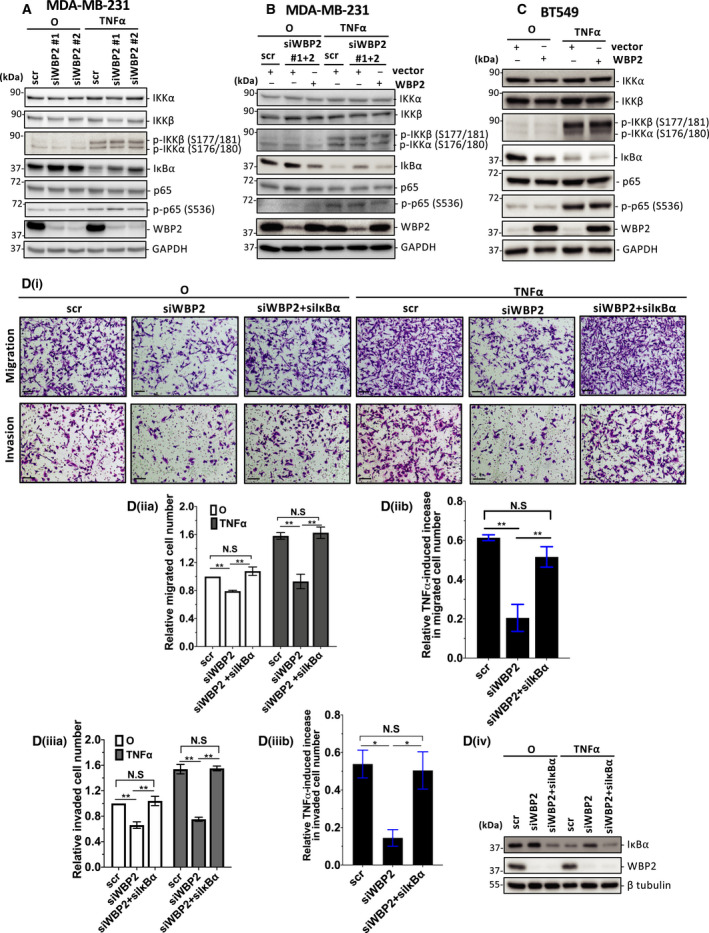
WBP2 limits IκBα protein level to drive TNF‐α‐induced migration and invasion. (A–C) Western blot analysis of NF‐κB signaling components upon 15min of TNF‐α stimulation (A) WBP2 was knocked down in MDA‐MB‐231 cells using two different siRNAs (siWBP2#1 and siWBP2#2) or scrambled (scr) siRNA control. (B) MDA‐MB‐231 cells were cotransfected with either scrambled (scr) or pooled siWBP2 (siWBP2#1 + 2), along with vector or WBP2‐expressing plasmid. (C) BT549 cells were transfected with either WBP2‐expressing plasmid or vector control. For A‐C, GAPDH was probed as a loading control. (D) Diminished cell migration and invasion caused by loss of WBP2 expression were reverted by simultaneous loss of inhibitor of nuclear factor of kappa beta alpha (IκBα). (i) Representative images of migrated and invaded MDA‐MB‐231 cells. Cells from 10 random fields of each sample were imaged. Magnification: 10×, scale bar (black): 250 µm. (ii) (a) Relative number of migrated cells were quantified and normalized to unstimulated scr control. O annotate cells unstimulated with TNF‐α. (ii) (b) Fold increase in migrated cells induced by TNF‐α. (iii) (a) Relative invaded cell number was quantified and normalized to unstimulated scr control (iii) (b) Fold increase in invaded cells induced by TNF‐α. Data are represented as mean ± SEM, *n* = 3. *P < 0.05; **P < 0.01 (one‐way ANOVA followed by *post hoc* Bonferroni test). (iv) WBP2 and IκBα knockdown in was validated in Western blot. β‐Tubulin was used as a loading control.

TNF‐α is known to mediate two main signaling pathways, NF‐κB and JNK pathways [49]. Our group has previously reported that WBP2 can activate JNK pathway in breast cancer [26]; however, the involvement of WBP2 in NF‐κB signaling has not been explored. Additionally, our above proteogenomic analysis and previous RNA‐Seq data [26] demonstrated a number of inflammatory pathways associated with WBP2 expression, in which NF‐κB is a key mediator [6]. Firstly, we sought to investigate whether WBP2 can mediate TNF‐α‐induced NF‐κB activity in TNBC. The NF‐κB reporter assay was performed to evaluate the NF‐κB transcriptional activity in three different TNBC cell lines. Shown in Fig. 4A, WBP2 silencing in high‐WBP2‐expressing MDA‐MB‐231 and MDA‐MB‐468 reduced NF‐κB‐dependent reporter activity, especially in TNF‐α‐stimulated conditions. Conversely, elevated expression of WBP2 in low WBP2‐expressing BT549 cells drove NF‐κB reporter activity in both basal condition and TNF‐α stimulation (Fig. 4A). Consistent with the above data, WBP2 knockdown significantly reduced TNF‐α‐induced nuclear localization of NF‐κB p65 subunit, a well‐known subunit of NF‐κB, as demonstrated by the reduced nuclear: cytoplasmic ratio of p65 upon TNF‐α stimulation in Fig. 4Bi‐ii. Regulation of p65 cellular localization by WBP2 is unlikely to be due to modulation of the total cellular p65 expression (Fig. 4Biii). WBP2 is known for its transcriptional coactivation activity in Wnt, ER/PR, and Hippo signaling and found to migrate into the nucleus upon ligand stimulation by Wnt or E2 [17, 22, 26]. Hence, we investigated whether TNF‐α can induce nuclear entry of WBP2 along with NF‐κB p65. Subcellular fractionation experiment demonstrated that TNF‐α induced p65 nuclear expression (Fig. 4Ci,iii) but did not significantly alter WBP2 nuclear expression (Fig. 4Ci,ii). Next, we hypothesized that WBP2 may act upstream of p65 to induce nuclear localization of the latter. An examination of upstream NF‐κB signaling components such as IKKs and IκBα revealed a negative correlation between WBP2 expression and IκBα protein levels in both basal and TNF‐α‐stimulated conditions (Fig. 5A–C and Fig. S4). Expression and phosphorylation of upstream IKKs, as well as downstream p65, remained unaltered upon WBP2 manipulation (Fig. 5A–C). Hence, it is conceivable that WBP2 negatively regulates IκBα abundance. Furthermore, abrogation of IκBα restored the impairment in migrative and invasive ability caused by WBP2 silencing both in the absence and in the presence of TNF‐α induction (Fig. 5Di–iv). Collectively, WBP2 is likely to drive TNF‐α‐induced TNBC migration and invasion via downregulating IκBα protein abundance in TNBC cells.

### 3.5 WBP2 promotes IκBα proteasomal degradation via modulating IκBα ubiquitination

**Fig. 6:**
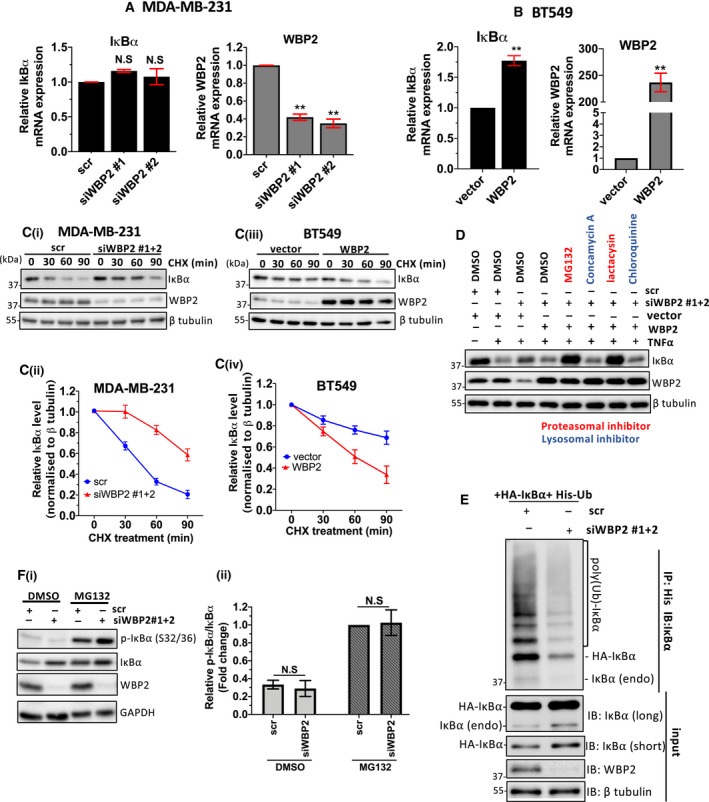
WBP2 promotes ubiquitin‐mediated IκBα proteasomal degradation through enhancing BTRC expression. (A, B) Analysis of inhibitor of nuclear factor of kappa beta alpha (IκBα) transcript levels in (A) MDA‐MB‐231 and (B) BT549 cells. WBP2 transcript level was also assessed to confirm efficiency of WBP2 knockdown or overexpression. Data are represented as mean ± SEM, *n* = 3. **P < 0.01. (C) Cycloheximide chase assay to assess WBP2 effect on IκBα stability. (i) Western blot analysis of IκBα in MDA‐MB‐231 transfected with scrambled (scr) siRNA or pooled WBP2 siRNAs (equal amounts of siWBP2#1&2) and (iii) BT549 transfected with WBP2‐expressing plasmid or vector control. The cells were treated with cycloheximide (CHX) for a duration of up to 90min. β‐Tubulin served as a loading control. (ii, iv) Quantification of IκBα bands in C normalized to β tubulin in (ii) MDA‐MB‐231 and (iv) BT549 cells using imagej software. (D) MDA‐MB‐231 was transfected with scr/pooled WBP2 siRNAs and vector/WBP2 plasmids, as indicated. The cells were serum‐starved overnight and then treated with the indicated proteasomal (red)/lysosomal (blue) inhibitors or DMSO control for 4–6 h before being stimulated with 10 ng·mL^−1^ of tumor necrosis factor alpha (TNF‐α) for 15 min. (E) *In vivo* ubiquitination assay performed in MDA‐MB‐231. Cells were treated with MG132 for 4–6 h before His pulldown was performed to immunoprecipitate the ubiquitinated proteins. The total amount of ubiquitinated IκBα was probed in Western blot. (F) WBP2‐silenced MDA‐MB‐231 cells were treated with MG132 (20 μm) for 4–6 h. (i) Western blot analysis was performed to probe the total and phosphorylated IκBα proteins (ii) Relative IκBα phosphorylation was quantified by normalizing phosphorylated IκBα to total IκBα via densitometry analysis. All data in this figure are represented as mean ± SEM, *n* = 3. **P < 0.01; N.S, nonsignificant. (unpaired *t*‐test for two group comparisons; one‐way ANOVA followed by *post hoc* Bonferroni test for multiple group *t*‐test).

Since WBP2 could mediate IκBα protein abundance even without TNF‐α stimulation as observed in Fig. 5A–C, we decided to examine the direct effects of WBP2 on IκBα without administering TNF‐α. Analysis by qPCR demonstrated that WBP2 silencing did not significantly alter IκBα gene expression in MDA‐MB‐231 (Fig. 6A). However, overexpression of WBP2 significantly raised IκBα transcript levels in BT549 (Fig. 6B). The enhanced IκBα mRNA expression in WBP2‐overexpressed BT549 is contradictory to the lower protein abundance observed in Fig. 5C. This could be a result of enhanced IκBα transcription by the putative WBP2‐induced NF‐κB activation since IκBα is known to be a transcription target of NF‐κB [57]. Nevertheless, the data imply that the negative regulation of WBP2 on IκBα is unlikely due to transcriptional mechanism.

Post‐translational modification is a pivotal mechanism controlling IκBα protein stability. Cycloheximide chase assay revealed that WBP2 reduces IκBα protein stability (Fig. 6Ci–iv). Furthermore, treatment with proteasomal inhibitors, and not lysosomal inhibitors, was sufficient to abrogate WBP2‐driven IκBα downregulation (Fig. 6D). To confirm that WBP2‐mediated IκBα degradation is mediated by polyubiquitination, the cells were pretreated with proteasomal inhibitor, MG132, to avert degradation of ubiquitinated IκBα proteins. The ubiquitinated proteins were then immunoprecipitated and probed for IκBα via immunoblotting. Figure 6E revealed that WBP2 silencing limited ubiquitinated IκBα polyubiquitination.

Phosphorylation of IκBα at serine 32 and 36 is crucial for the recognition of upstream E3 ligase to mediate ubiquitination. Therefore, we questioned if WBP2 mediates IκBα ubiquitination by promoting IκBα phosphorylation. TNF‐α was added to the cells to induce IκBα phosphorylation. WBP2 silencing or overexpression did not significantly alter the proportion of phosphorylated IκBα when normalized to total IκBα expression (Fig. S5A,B). To validate that the actual IκBα phosphorylation status is not masked by TNF‐α‐mediated degradation, the cells were treated with MG132 to stabilize IκBα and enable accumulation of phosphorylated IκBα. Again, the level of phosphorylated IκBα was not significantly altered with WBP2 downregulation (Fig. 6Fi,ii). Therefore, WBP2 downregulates IκBα expression via ubiquitin‐mediated proteasomal degradation.

### 3.6 WBP2 promotes mRNA stability of BTRC, a component of E3 ligase for IκBα

**Fig. 7:**
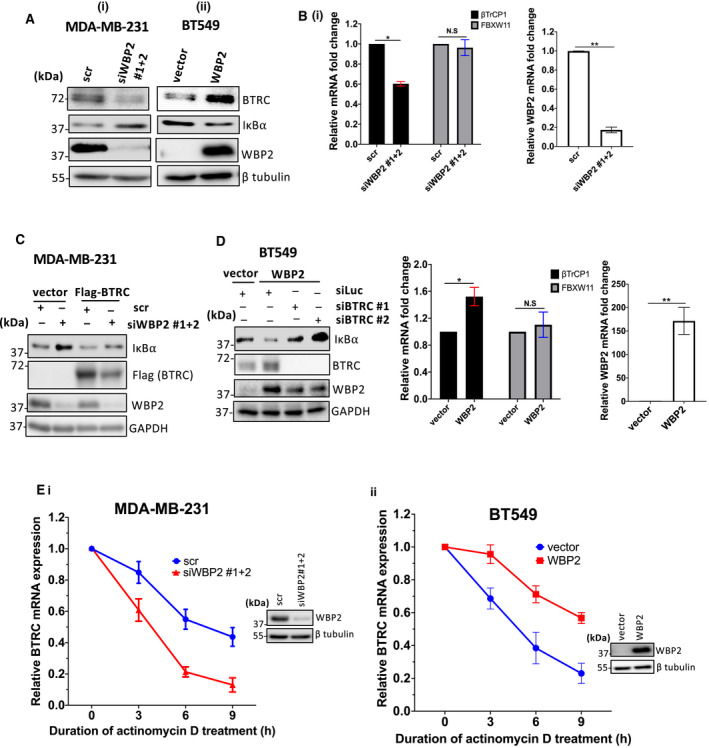
WBP2 upregulates *BTRC* expression through inducing *BTRC* mRNA stability. (A) Immunoblots showing the protein expression of beta‐transducin repeat containing E3 ubiquitin ligase (BTRC) upon (i) WBP2 knockdown in MDA‐MB‐231 and (ii) WBP2 overexpression in BT549 cells. β‐Tubulin is the loading control. (B) Quantification of βTrCP1 (BTRC) and F‐Box/WD repeat‐containing protein 11 (FBXW11) transcripts in (i) WBP2‐silenced MDA‐MB‐231 cells and (ii) WBP2‐overexpressed BT549 cells. WBP2 knockdown and overexpression were also validated via qPCR. Data are represented as mean ± SEM, *n* = 3. N.S, nonsignificant; *P < 0.05, **P < 0.01 (unpaired *t*‐test). (C) Representative immunoblot indicating that BTRC overexpression reverted IκBα stability in WBP2‐silenced MDA‐MB‐231 cells, *n* = 3. (D) Representative immunoblot from indicating that BTRC deficiency restored IκBα protein abundance in WBP2‐overexpressed BT549 cells, *n* = 3. (E) Actinomycin D assay performed on (i) WBP2 knockdown MDA‐MB‐231 and (ii) WBP2‐overexpressed BT549. Cells were treated with actinomycin D for up to 9h. The mRNA levels of BTRC were quantified via quantitative PCR and normalized to 18S. Data are represented as mean ± SEM, *n* = 3. Western blot confirmed knockdown and overexpression of WBP2 at time point of 0 h.

βTrCP is the E3 ligase for IκBα [8, 9]. It consists of two paralogs, BTRC (also βTrCP1) and FBXW11 (also βTrCP2). We speculate that WBP2 modulates BTRC to influence IκBα ubiquitination. Through immunoblotting, WBP2 silencing in MDA‐MB‐231 cells ameliorated BTRC protein expression (Fig. 7Ai), while the converse is true in WBP2‐overexpressed BT549 cells (Fig. 7Aii). The doublet seen in BTRC protein in Fig. 7Ai,ii is likely due to the alternative splicing isoforms, with a difference in around 35 amino acids [58]. Next, we assessed whether WBP2 affects the transcript levels of BTRC and FBXW11 using primers specific to either BTRC or FBXW11. WBP2 expression was shown to be concomitant to BTRC transcript abundance, but not FBXW11 transcripts (Fig. 7Bi,ii). To ascertain that WBP2 mediates IκBα downregulation through regulating BTRC expression, exogeneous BTRC was introduced into WBP2 knockdown MDA‐MB‐231 cells. Elevated BTRC expression was sufficient to subvert IκBα upregulation mediated by WBP2 knockdown (Fig. 7C). On the other hand, elevated WBP2 expression in BT549 cells led to a drop in IκBα expression, which can be reversed by simultaneously silencing BTRC expression (Fig. 7D). A slight downregulation of exogeneous WBP2 levels was observed in BTRC‐silenced BT549. Intriguingly, overexpression of BTRC in MDA‐MB‐231 did not drive WBP2 expression in MDA‐MB‐231 (Fig. 7C). The apparent discrepancy between the observations obtained via loss‐ and gain‐of‐function studies remains to be investigated. Overall, these findings suggest that BTRC is crucial for WBP2‐induced IκBα downregulation in TNBC.

The mRNA of BTRC is highly unstable and can be subjected to posttranscriptional regulation [59, 60, 61]. Since WBP2 can induce BTRC mRNA levels, we postulated that WBP2 negatively modulates BTRC mRNA stability. Treatment with actinomycin D, a transcription inhibitor, revealed that WBP2 reduced the half‐life of BTRC mRNA (Fig. 7Ei,ii). Hence, it is conceivable that WBP2 influences BTRC mRNA stability via posttranscriptional modifications.

### 3.7 BTRC predicts poorer patient survival and is crucial for WBP2‐driven TNBC cell migration and invasion

**Fig. 8:**
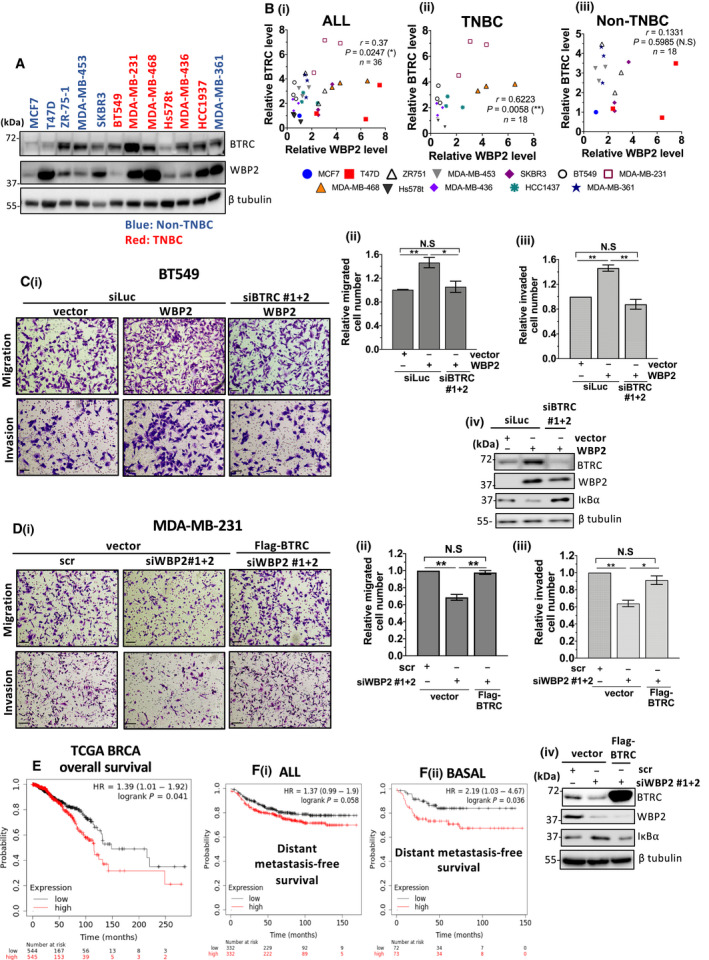
BTRC is crucial for WBP2‐driven TNBC migration and invasion, and WBP2/BTRC/IκBα is linked to poorer clinical prognosis. (A) Representative immunoblot indicating coexpression of beta‐transducin repeat containing E3 ubiquitin ligase (BTRC) and WBP2 protein levels in a screen of 12 breast cancer cell lines. The non‐TNBC cell lines are indicated in blue, while TNBC cell lines are indicated in red. (B) Scatter plot indicating the correlation between WBP2 and BTRC in (i) all cell lines (include both TNBC and non‐TNBC) (ii) non‐TNBC cell lines and (iii) TNBC cell lines. The expression of BTRC and WBP2 was measured thrice in all cell lines, except MDA‐MB‐361 which was repeated twice. Each data point represents one experimental replicate and is color‐coded according to its cell line. WBP2 and BTRC expression of all cell lines from (A) is measured relative to MCF7 via imagej. Three experimental replicates were performed for all cell lines and indicated in the scatter plot. *P < 0.05, **P < 0.01 (Spearman's correlation test). (C) BTRC reduction abolished WBP2‐driven BT549 migration and invasion cells. (D) BTRC overexpression rescued impairment in cell migration and invasion in WBP2‐depleted MDA‐MB‐231 cells. (C, D) (i) Representative images from Transwell migration and invasion assays. Cells from 10 random fields of each sample were imaged. Magnification: 10×, scale bar (black line on bottom left): 250 µm, (ii&iii) Relative number of (ii) migrated and (iii) invaded cells was quantified via imagej. Error bars indicate the SEM, *n* = 3. *P < 0.05 **P < 0.01 (one‐way ANOVA followed by *post hoc* Bonferroni test). (iv) Western blot was performed to validate WBP2 and BTRC expression. (E) Kaplan–Meier plot showing overall survival for TCGA BRCA patient. The mean WBP2, BTRC, and inverted IκBα gene expression was computed from KM plotter. All genes were given the same weightage, and only IκBα values were inverted. The TCGA BRCA patients were stratified into two groups by the median gene signature. (F) Kaplan–Meier plot showing distant metastasis‐free survival (DMFS) of (i) all breast cancer patients and (ii) basal breast cancer patients. Gene expression data and disease‐free and overall survival information are from GEO. Patient samples were split into two groups according to the median of the gene signature (mean WBP2, BTRC, and inverted IκBα gene expression computed from KM plotter). During analysis, no restriction was placed on tumor, node, metastasis classification system result, Lauren classification, gender, or treatment.

Given that WBP2 promotes BTRC expression, we investigated their coexpression in a breast cancer cell line panel (Fig. 8A). WBP2 expression was positively and significantly correlated with BTRC, with a moderate correlation coefficient of > 0.39, in all cell lines (Fig. 8Bi). However, this correlation coefficient was much higher in TNBC cell lines (*r* = 0.6223) (Fig. 8Bii). On the other hand, the correlation between WBP2 and BTRC expression in non‐TNBC cell lines was not statistically significant (Fig. 8Biii). Therefore, our findings concur with the notion that WBP2 is positively associated with BTRC in breast cancer, especially in TNBC.

BTRC is proposed to play dual roles in cancers, displaying oncogenic properties in one context and tumor‐suppressive characteristics in another [62, 63, 64]. To determine whether BTRC protein expression predicts poorer prognosis in clinical breast cancer, Kaplan–Meier meta‐analysis was performed on the breast cancer cohort in Tang *et al*. [65] via public KM plotter. Median value based on BTRC mass spectrometry data was used to segregate the samples into two groups for analysis. Higher BTRC protein expression significantly reduced overall patients' survival, especially in ER‐negative tumors (Fig. S6). Therefore, the result in Fig. S6 supports the notion that BTRC is an oncogene in ER‐negative breast cancer.

With consideration of the tumor‐promoting role of BTRC, and the positive regulatory role of WBP2 on BTRC, we questioned whether WBP2 mediates breast cancer migration and invasion via BTRC. As expected, loss of BTRC expression diminished WBP2‐induced cell migration and invasion in BT549 and relative migrated and invaded cell number was negatively correlated with IκBα abundance (Fig. 8Ci–iv). Consistently, overexpression of BTRC in MDA‐MB‐231 cells was sufficient to restore the impairment in migration and invasion mediated by WBP2 knockdown (Fig. 8Di–iv). Together, these *in vitro* findings led us to the hypothesis that elevated WBP2 and BTRC expression, coupled with reduced IκBα expression, predicts poorer prognosis for clinical breast cancer. To test this hypothesis, the mean expression of WBP2, BTRC, and inverted IκBα values was analyzed on TCGA BRCA in KM Plotter. A mean gene signature was generated by giving all genes the same weightage and inverting IκBα expression, since IκBα is negatively regulated by WBP2 and BTRC. A high gene signature implies higher *WBP2* and *BTRC* gene expression, combined with lower *IκBα* gene expression, and vice versa. Our results demonstrated that higher *WBP2/BTRC/IκBα* gene signature significantly correlates with lower overall survival in TCGA BRCA (Fig. 8E). Additionally, a survey of public microarray data repositories for survival among 5667 breast cancer patients in KM plotter database demonstrated that elevated WBP2/BTRC/IκBα gene signature is significantly correlated to distant metastasis‐free survival (DMFS) with a hazard ratio (HR) of 1.37 (Fig. 8Fi). The hazard ratio was much higher (HR = 2.19) in basal breast cancer, which predominantly composes of TNBC (Fig. 8Fii). Therefore, the *WBP2/BTRC/IκBα* signaling axis was associated with poorer survival outcomes in clinical breast cancer, especially in basal breast cancer, in congruence with the findings presented in this study. Collectively, WBP2 is likely to drive TNF‐α‐induced TNBC cell migration and invasion through the BTRC/IκBα signaling axis.

## 4 Discussion

The expression and activity of oncogenes and tumor suppressors can be modulated via multiple levels of regulation. Previous studies have arrived at differing conclusions for the degree of concordance between copy number variation, gene, and protein expression in tumor‐promoting or tumor‐suppressive markers [66, 67]. In this study, we examined the correlation between *WBP2* copy number variation, gene, and protein expression. We observed only at best partial to low concordance between *WBP2* copy number and gene expression, as well as between gene and protein levels. This is consistent with previous studies reported by Li *et al*. [26] and Tabatabaeian *et al*. [42] Consequently, we surmised that the examination of WBP2 protein expression, rather than gene expression or copy number variation, is more likely to give an accurate prediction of the molecular roles of WBP2 in cancers. These observations, coupled with the fact that tumor biology tends to be directly affected by protein function, illustrated the pressing need to adopt an integrated proteogenomic approach in the study of tumor development.

In recent years, publicly available mass spectrometry‐based proteomic data on selected clinical samples from TCGA dataset from Clinical Proteomic Tumor Analysis Consortium (CPTAC) opened up possibilities for researchers to explore global proteome in large‐scale cancer cohorts [68, 69, 70, 71]. Integration of tumor proteomic and genomic analysis provided a fresh approach for the discovery of new biological insights and novel therapeutic targets [69, 70]. In this study, we tapped into the potential of integrated proteogenomic analysis to delineate the sophisticated signaling network modulated by cancer oncogene, particularly WBP2, in TCGA BRCA. Although the current dataset is only constrained to 74 samples due to limited available WBP2 mass spectrometry‐based expression, many of the signaling pathways revealed in our proteogenomic integration have been reported to be regulated by WBP2 in other papers, indicating a high accuracy and reliability of our analysis workflow. Our study exemplified for the first time how proteogenomic integration can be utilized as an unbiased and data‐driven approach to unearth novel and previously unchartered mechanistic insights behind a solitary oncogene in a large‐scale clinical setting. Several pathways, such as cadherin, Alzheimer's disease, and angiogenesis signaling, highlighted to be associated with WBP2 in this proteogenomic study have yet been explored.

TNF‐α is a central regulator of inflammation. Given the link between inflammation and cancer, it is not unexpected that an increasing number of studies documents TNF‐α as a tumor promoter in breast cancer [46, 47, 48, 49, 50, 72, 73, 74], supporting the notion that TNF‐α is important for breast cancer progression. Here, we demonstrated that TNF signaling components are likely to be enriched in TNBC or basal cell lines compared with their other counterparts from other subtypes, implying that TNF signaling expression may be linked to breast cancer aggressiveness. This corroborates with previous study that suggested that TNF signaling is enriched in basal breast cancer than nonbasal breast tumors, along with other inflammatory pathways such as cytokine–cytokine receptor interaction and Toll‐like signaling [75]. Although the physiological TNF‐α serum concentration is in pg·mL^−1^ range, the concentration is much elevated in cancer patients and can reach up to ng·mL^−1^ range [76]. Therefore, the concentration of 10 ng·mL^−1^ of TNF‐α used in this study mimicked the proinflammatory environment in cancer condition. Additionally, 10–100 ng·mL^−1^ TNF‐α is commonly used for *in vitro* experiments [48, 51, 77, 78].

Despite the tumor‐promoting role of TNF‐α in breast cancer, only a handful of studies have attempted to decipher the mechanism of TNF‐α in driving breast cancer progression, and NF‐κB appeared to be an integral component of TNF‐α‐induced breast cancer aggressiveness [46, 78, 79]. In this study, it is evident that WBP2 promotes TNF‐α‐induced TNBC aggressive phenotypes including migration and invasion through negatively regulating IκBα. Since the main mode of mechanism of WBP2 on TNF‐α‐induced migration is through IκBα, and many inflammatory mediators such as IL1, IL6 and LPS can induce IκBα degradation, we expect WBP2 to be able to regulate NF‐κB pathway upon stimulation with other inflammatory factors. While not all breast cancers exhibit inflammatory microenvironment, TNBC is the molecular subtype associated with the worst prognosis and a proinflammatory microenvironment [9, 10]. Furthermore, NF‐κB was reported to be constitutively activated in TNBC compared with other molecular subtypes and is believed to contribute to poorer prognosis in TNBC [6, 7, 8]. Therefore, we envisage that our study will provide new insights into therapeutic targets for inflammation‐driven TNBC. On the other hand, since TNF and NF‐κB signaling have been reported to affect ER+ and HER2+ breast cancer. It will be interesting to investigate whether the regulatory role of WBP2 on NF‐κB activation can also be observed in non‐TNBC.

Interestingly, TNF‐α did not stimulate upregulation of WBP2 nuclear expression, as in the case of Wnt or E2 stimulation. Therefore, we postulated that WBP2 may not act as a transcriptional coactivator for p65 to drive NF‐κB signaling, unlike in the case of ER and Wnt signaling. This finding therefore marks the first time that WBP2 can control the stability of another protein. A more in‐depth analysis shows that WBP2 modulates the expression of BTRC, the E3 ligase component specific for recognizing IκBα. Because BTRC can either act as a tumor suppressor or promoter in various cancers [62, 63, 64], we validated its role in breast cancer by performing Kaplan–Meier survival analysis based on its protein expression. Our results showed that BTRC acts as an oncogene in breast cancer. Functional studies confirmed that BTRC is essential for WBP2's stimulatory effect on cell migration. Consistently, we observed that combinatorial gene expression of WBP2, BTRC, and inverted IκBα associates with poorer prognosis in clinical breast cancer patients. Together, all these findings provided evidence that WBP2 promotes breast cancer by downregulating IκBα through driving BTRC expression. The data in this study add a new dimension to the multimodal actions of WBP2 previously reported to drive TNBC progression, including Hippo and Wnt signaling, where WBP2 functions primarily as a transcriptional coactivator for YAP and β catenin [17, 24]. Furthermore, WBP2 has been recently shown to associate with LATS2 and WWC3 to inhibit the Hippo tumor suppressor pathway in gastric and lung cancers, in turn limiting cancer proliferation and invasion [20, 21]. However, one limitation of our study is the lack of *in vivo* experiments to support our proposed mechanism. Nevertheless, our group has previously reported that WBP2 silencing diminished tumor growth in TNBC xenografts [17, 25]. A substantial evidence has highlighted the role of WBP2 in metastasis in HER2‐positive breast cancer and lung carcinoma *in vivo* [20, 80]. It would be of interest to validate the role of WBP2 in TNBC metastasis *in vivo* for future studies.

Interestingly, a closer investigation on the mode of action of WBP2 on BTRC revealed that WBP2 likely regulates BTRC via posttranscriptional modifications. Coincidentally, BTRC mRNA is predominantly regulated by miRNAs through JNK and Wnt pathways [59, 60, 61]. Although the exact mechanism of WBP2 on BTRC mRNA stability is not fully elucidated in this study, it is reasonable to speculate that this observation could be due to the reported involvement of WBP2 in JNK and Wnt signaling [17, 18, 26]. Recently, our group discovered that WBP2 may negatively regulate the activity of microprocessor complex, perhaps through the binding to the WW domain of DGCR8, a critical component of the microprocessor complex [81]. It is conceivable that WBP2 could be involved in the control of mRNA stability of oncogenes other than BTRC to drive cancer biology.

## 5 Conclusion

In conclusion, we demonstrated that WBP2 drives TNBC migration and invasion under TNF‐α‐induced conditions, which mimic the tumor‐stimulatory microenvironment. Although previous studies hinted the possible involvement of WBP2 in inflammation, via its binding to ITCH [17, 82], a negative regulator of TNF/NF‐κB signaling, this study shows for the first time a participatory role of WBP2 in tumor inflammatory signaling, particularly in TNF/NF‐κB signaling pathway. Moreover, its regulation on BTRC mRNA stability highlights an alternative mode of action of WBP2 as a posttranscriptional regulator, besides its widely reported functional role as a transcriptional coactivator.

## Conflict of interest

The authors declare no conflict of interest.

## Author contributions

YXL and YPL conceived the study concept; YPL provided the direction, scientific inputs, and editorial advice; YXL and YPL designed the experiment; YXL performed the experiment; YXL, HL, and TC analyzed the data. YXL wrote the first draft of manuscript. HL and TC reviewed and edited the manuscript. All authors discussed the results, provided critical feedback, and helped shape the research, analysis, and manuscript.

### Peer Review

The peer review history for this article is available at https://publons.com/publon/10.1002/1878‐0261.13048.

## Supporting information


**Fig. S1.** Correlation between *WBP2* copy number and mRNA z‐scores in individual tumor types. Scatter plot showing correlation between WBP2 copy number in TCGA LIHC, TCGA OV, TCGA UCS, TCGA COADREAD, TCGA AML and TCGA UVM. The left panel (LIHC, OV, and UCS) represents tumor types with high frequencies of WBP2 copy number amplification; while the right panel (COADREAD, UVM and AML) represents the tumor types with low or no WBP2 amplification. Each dot represents an individual sample, and the samples are colored according to their *WBP2* copy number alterations. Spearman's correlation test was performed. ***p < 0.001, N.S non‐significant.

**Fig. S2.** Merged gene expression of TNF signaling components genes across different molecular subtypes in breast cancer cell line panel (A)(i) Box plot showing expression of TNF signaling merged gene set in cell lines grouped into Basal A (red), Basal B (green) and luminal (blue) or (ii) triple negative (TN, red), HER2‐ positive (HER2, purple) and hormone receptor positive (HR, blue). The range of the box is the inter‐quartile range for each tumor type, and the line in the box represents the median. (B) Expression of genes from TNF signaling gene set across 51 breast cancer cell lines. Colours according to (A). The number in brackets for x axis shows the number of cell lines associated with the molecular subtype. One‐way ANOVA test was performed to determine statistical significance. *** p < 0.001.

**Fig. S3.** WBP2 silencing reduced TNFα‐induced target gene expression. MDA‐MB‐231 cells were transfected with either siRNAs targeting WBP2 or scrambled (scr) siRNA. The cells were serum starved overnight and then treated with TNF⍺ for 6 h. RNA lysates were subjected to reverse transcription and qPCR to determine the transcriptional expression of TNF⍺‐induced genes*, IL1β, IL6, IL8, G‐CSF* and *MMP9*. *WBP2* mRNA expression was determined to confirm WBP2 knockdown in WBP2 siRNA‐transfected cells. All transcript quantification was normalized to 18S. Data are represented as mean ± SEM, n = 3. *p < 0.05, **p < 0.01 (one‐way ANOVA followed by post‐hoc Bonferroni test).

**Fig. S4.** Densitometry analysis indicating WBP2's effect on IκBα levels. IκBα protein expression in Fig. 5A–C was quantified and normalized to its loading control, β tubulin. Normalized IκBα levels were calculated in relative to scrambled (scr) siRNA/vector control in (A) MDA‐MB‐231 transfected with either siWBP2#1/2 or scr siRNA. (immunoblot shown in Fig. 5A), (B) MDA‐MB‐231 transfected with scr/pooled siWBP2#1 + 2, along with vector/WBP2 plasmids (immunoblot in Fig. 5B) and (C) BT549 cells transfected with vector or WBP2 plasmids. (immunoblot in Fig. 5C). All densitometry analysis was conducted using ImageJ. Data is represented as mean ± SEM, n = 3. *p < 0.05, ** p < 0.01 (one‐way ANOVA followed by post‐hoc Bonferroni test).

**Fig. S5.** WBP2 does not modulate TNFα‐induced IκBα phosphorylation. MDA‐MB‐231 was silenced with pooled siWBP2 #1 + 2. The cells were serum starved and then treated with TNFα for 15min. (i) Western blot analysis was performed to probe the total and phosphorylated IκBα proteins (ii) Relative IκBα phosphorylation were quantified by calculating phosphorylated IκBα in relative to total IκBα. The expression of total and phosphorylated IκBα was determined by densitometry analysis using ImageJ software. Data is represented as mean ± SEM, n = 3. *p < 0.05, ** p < 0.01 (one‐way ANOVA followed by post‐hoc Bonferroni test).

**Fig. S6.** BTRC is an oncogene in human breast cancer. Kaplan‐Meier analysis of breast cancer patients according to BTRC protein expression in all tumors, ER+ tumors and ER‐ tumors. Protein expression and clinical data of breast cancer cohort from Tang et al. (2018) [68] was obtained from KM plotter. The patients were split into two groups based on their median BTRC protein expression.

**Table S1.** List of siRNAs used in this study and their sequences.
**Table S2.** List of primers used in this study.
**Table S3.** Stratification of TCGA BRCA samples into WBP2p_high and WBP2p_low groups by their median WBP2 protein z‐scores.
**Table S4.** List of differentially enriched genes (DEGs) associated with WBP2 protein expression in TCGA BRCA.

## Data Availability

The data that support the findings of this study are available from the corresponding author (bchlyp@nus.edu.sg) upon reasonable request.
