# Supplementary figures and images for: WBP2 promotes BTRC mRNA stability to drive migration and invasion in triple‐negative breast cancer via NF‐κB activation
Source: Mol Oncol. 2021 Aug 12;16(2):422–46. doi: 10.1002/1878-0261.13048 (PMC8763649; doi:10.1002/1878-0261.13048)

Fig. S1

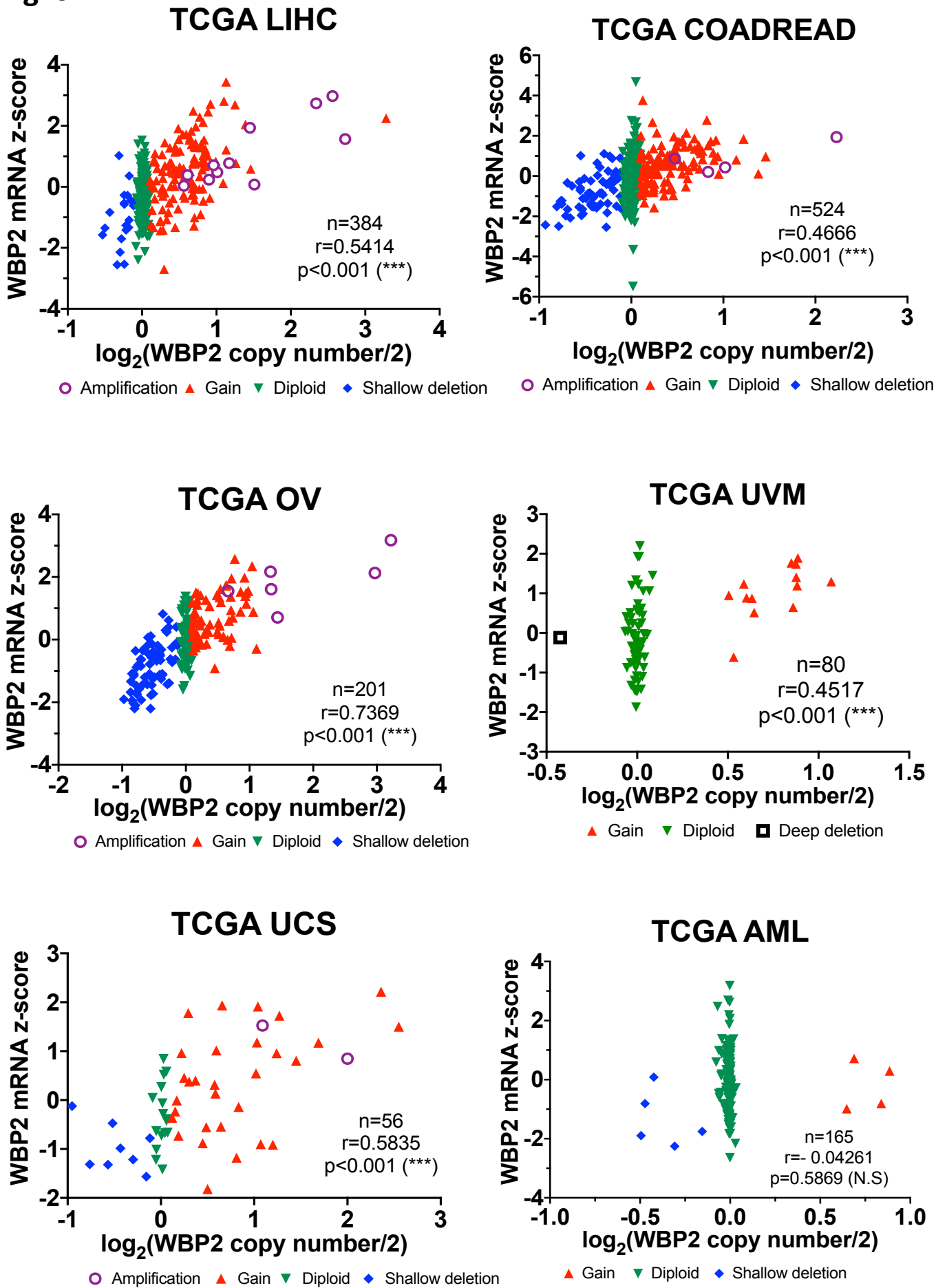

Supplement: Supplementary file 1 — Fig. S1. Correlation between WBP2 copy number and mRNA z‐scores in individual tumor types. Scatter plot showing correlation between WBP2 copy number in TCGA LIHC, TCGA OV, TCGA UCS, TCGA COADREAD, TCGA AML and TCGA UVM. The left panel (LIHC, OV, and UCS) represents tumor types with high frequencies of WBP2 copy number amplification; while the right panel (COADREAD, UVM and AML) represents the tumor types with low or no WBP2 amplification. Each dot represents an individual sample, and the samples are colored according to their WBP2 copy number alterations. Spearman's correlation test was performed. ***p < 0.001, N.S non‐significant. [file MOL2-16-422-s006.pdf]

Fig. S2

(A)(i)

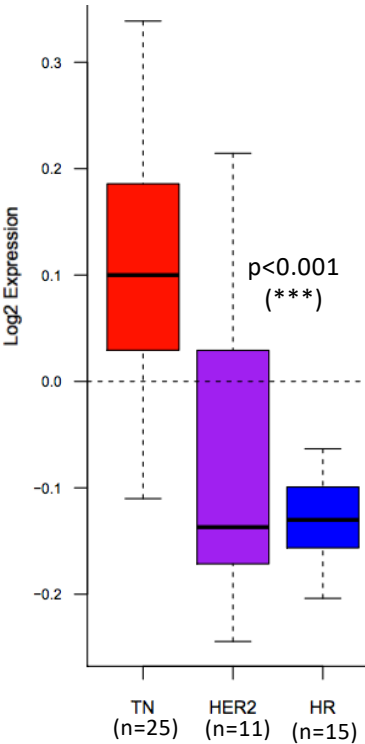

(ii)

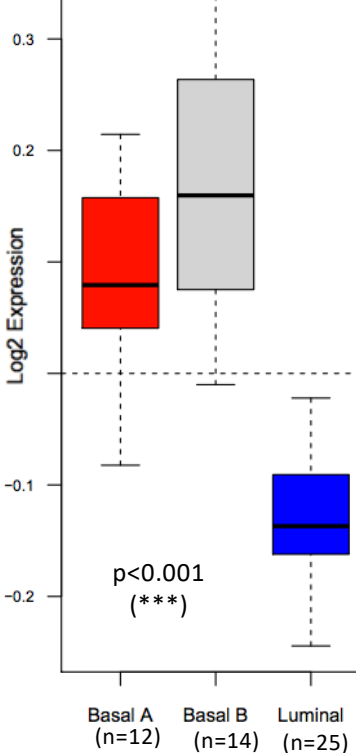

(B)

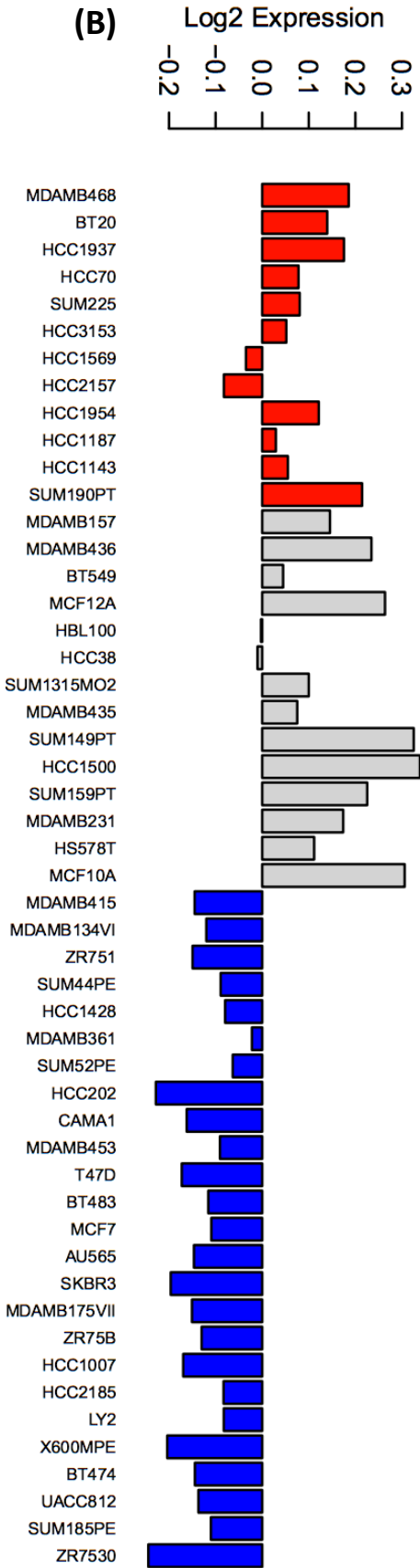

Supplement: Supplementary file 2 — Fig. S2. Merged gene expression of TNF signaling components genes across different molecular subtypes in breast cancer cell line panel (A)(i) Box plot showing expression of TNF signaling merged gene set in cell lines grouped into Basal A (red), Basal B (green) and luminal (blue) or (ii) triple negative (TN, red), HER2‐ positive (HER2, purple) and hormone receptor positive (HR, blue). The range of the box is the inter‐quartile range for each tumor type, and the line in the box represents the median. (B) Expression of genes from TNF signaling gene set across 51 breast cancer cell lines. Colours according to (A). The number in brackets for x axis shows the number of cell lines associated with the molecular subtype. One‐way ANOVA test was performed to determine statistical significance. *** p < 0.001. [file MOL2-16-422-s005.pdf]

Fig. S3

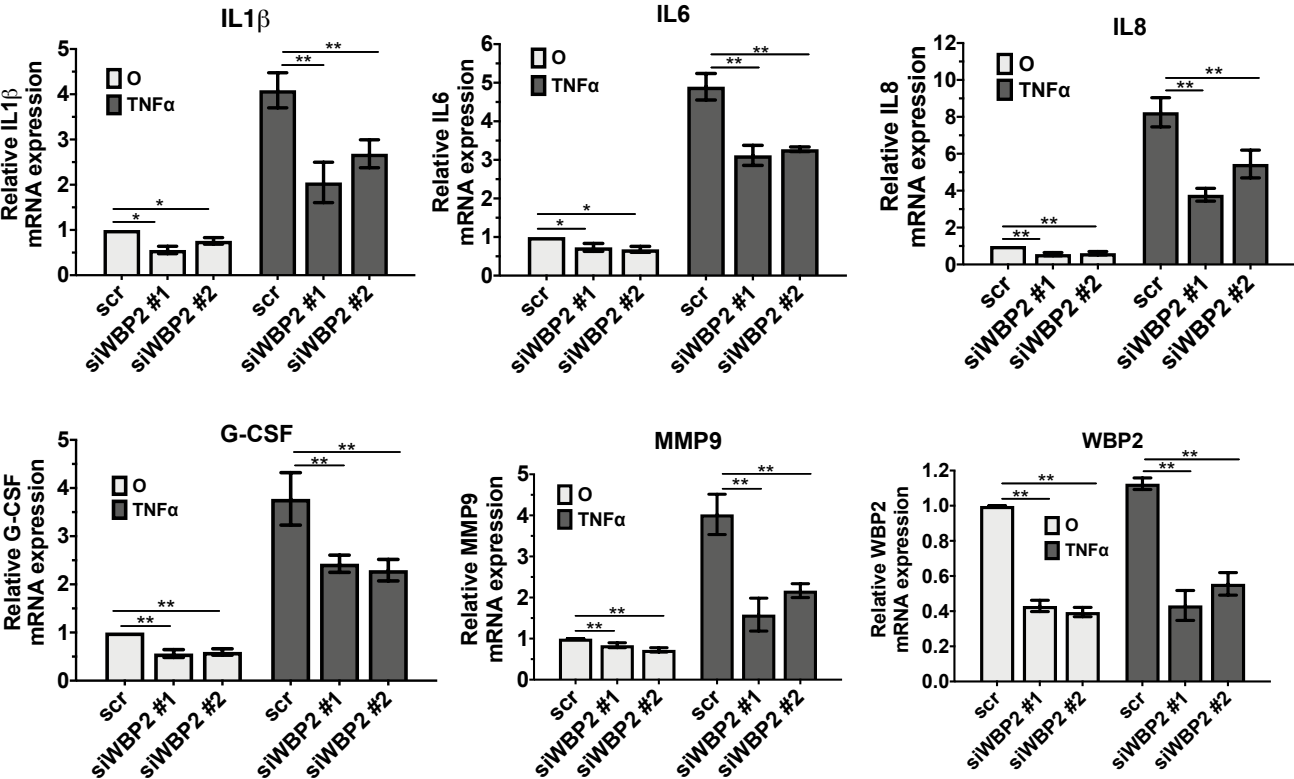

Supplement: Supplementary file 3 — Fig. S3. WBP2 silencing reduced TNFα‐induced target gene expression. MDA‐MB‐231 cells were transfected with either siRNAs targeting WBP2 or scrambled (scr) siRNA. The cells were serum starved overnight and then treated with TNF⍺ for 6 h. RNA lysates were subjected to reverse transcription and qPCR to determine the transcriptional expression of TNF⍺‐induced genes, IL1β, IL6, IL8, G‐CSF and MMP9. WBP2 mRNA expression was determined to confirm WBP2 knockdown in WBP2 siRNA‐transfected cells. All transcript quantification was normalized to 18S. Data are represented as mean ± SEM, n = 3. *p < 0.05, **p < 0.01 (one‐way ANOVA followed by post‐hoc Bonferroni test). [file MOL2-16-422-s007.pdf]

Fig. S4

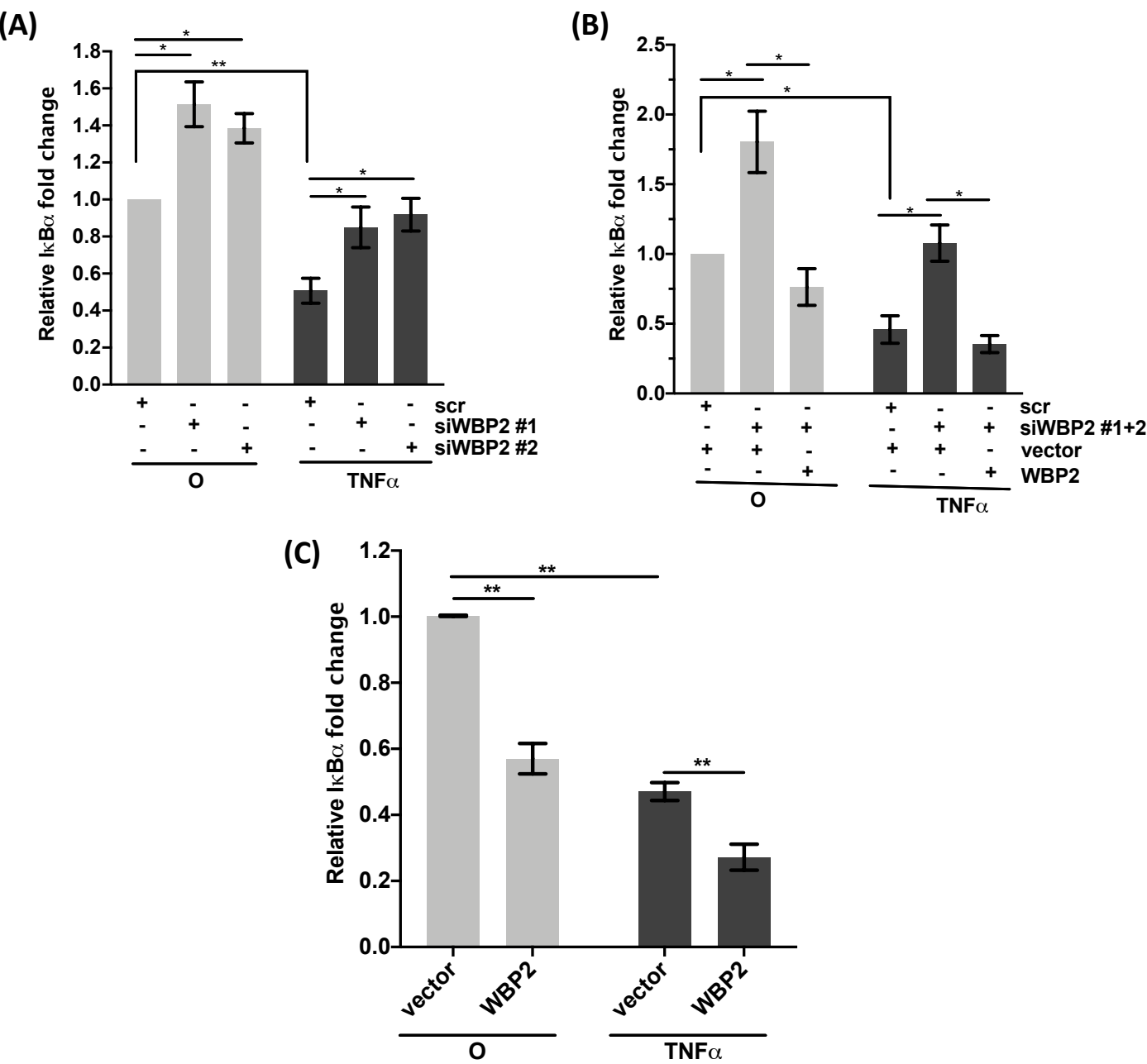

Supplement: Supplementary file 4 — Fig. S4. Densitometry analysis indicating WBP2's effect on IκBα levels. IκBα protein expression in Fig. 5A–C was quantified and normalized to its loading control, β tubulin. Normalized IκBα levels were calculated in relative to scrambled (scr) siRNA/vector control in (A) MDA‐MB‐231 transfected with either siWBP2#1/2 or scr siRNA. (immunoblot shown in Fig. 5A), (B) MDA‐MB‐231 transfected with scr/pooled siWBP2#1 + 2, along with vector/WBP2 plasmids (immunoblot in Fig. 5B) and (C) BT549 cells transfected with vector or WBP2 plasmids. (immunoblot in Fig. 5C). All densitometry analysis was conducted using ImageJ. Data is represented as mean ± SEM, n = 3. *p < 0.05, ** p < 0.01 (one‐way ANOVA followed by post‐hoc Bonferroni test). [file MOL2-16-422-s003.pdf]

Fig. S5

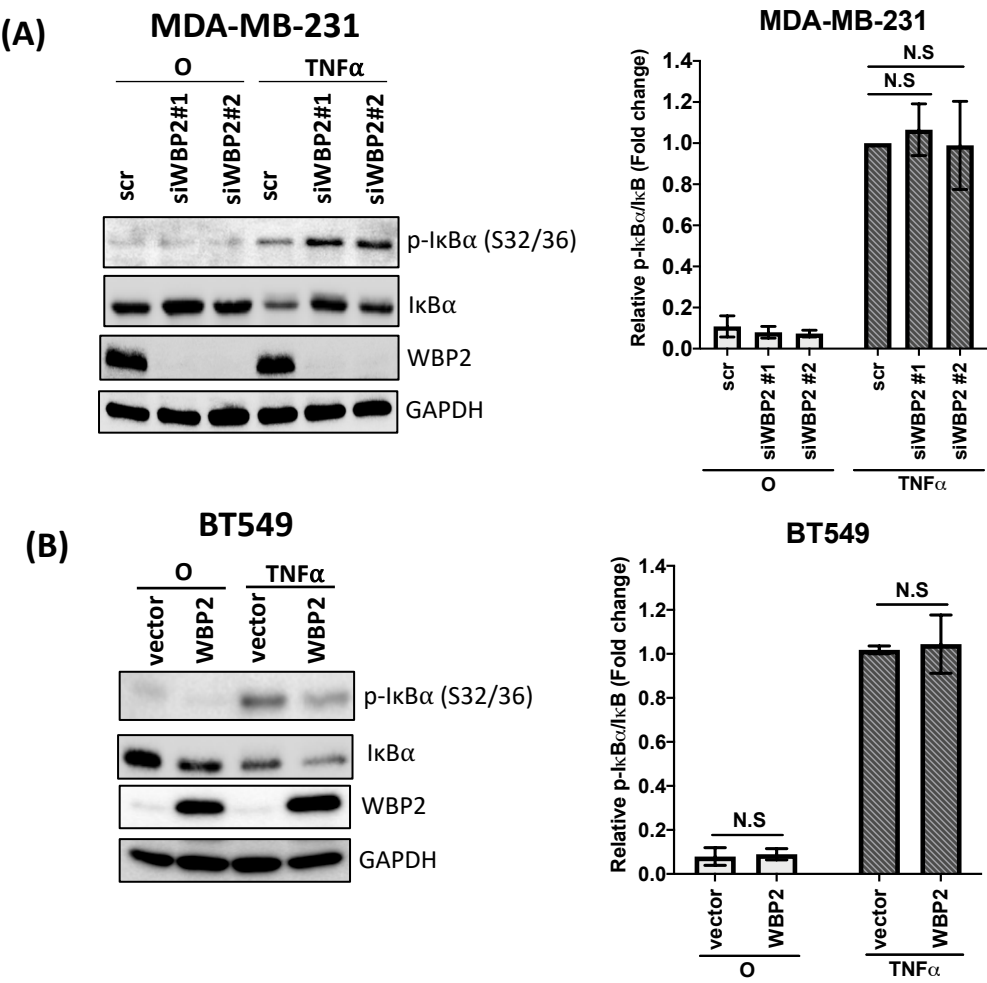

Supplement: Supplementary file 5 — Fig. S5. WBP2 does not modulate TNFα‐induced IκBα phosphorylation. MDA‐MB‐231 was silenced with pooled siWBP2 #1 + 2. The cells were serum starved and then treated with TNFα for 15min. (i) Western blot analysis was performed to probe the total and phosphorylated IκBα proteins (ii) Relative IκBα phosphorylation were quantified by calculating phosphorylated IκBα in relative to total IκBα. The expression of total and phosphorylated IκBα was determined by densitometry analysis using ImageJ software. Data is represented as mean ± SEM, n = 3. *p < 0.05, ** p < 0.01 (one‐way ANOVA followed by post‐hoc Bonferroni test). [file MOL2-16-422-s001.pdf]

Fig. S6

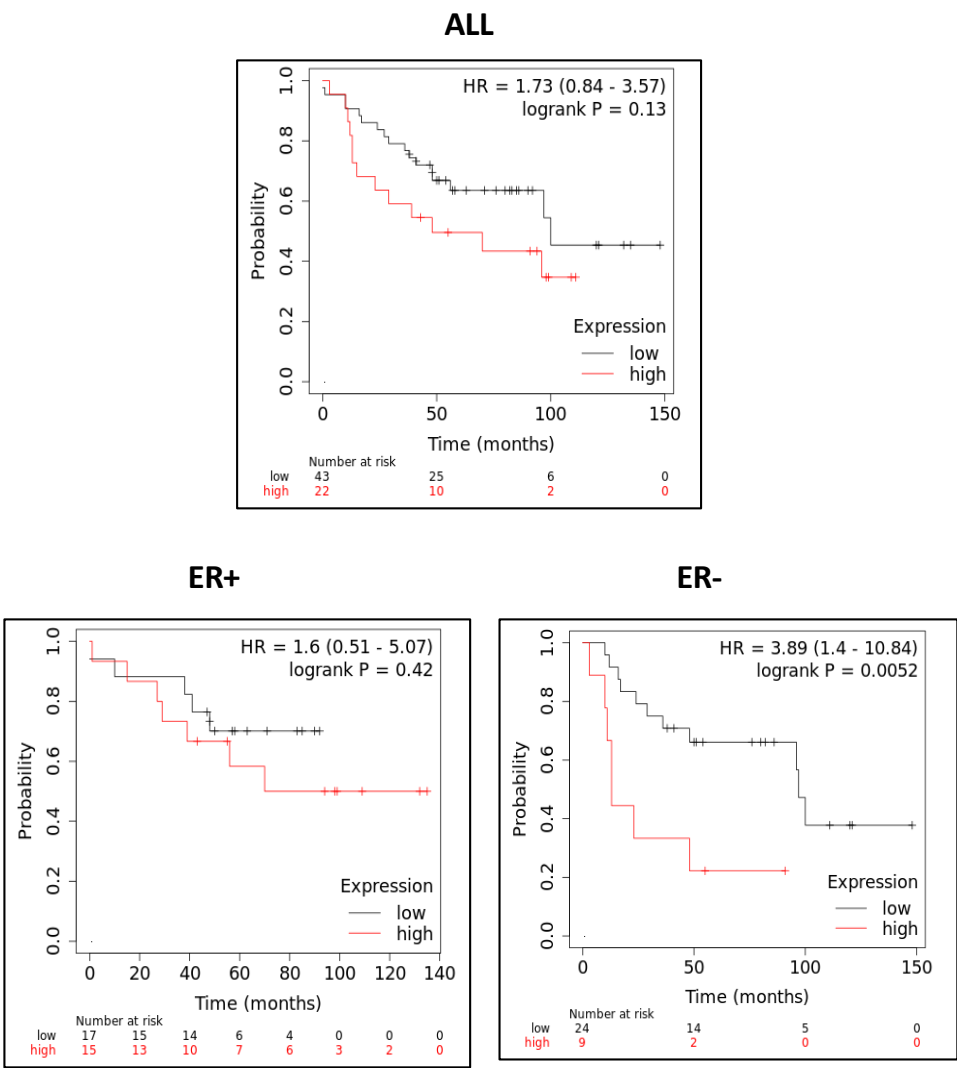

Supplement: Supplementary file 6 — Fig. S6. BTRC is an oncogene in human breast cancer. Kaplan‐Meier analysis of breast cancer patients according to BTRC protein expression in all tumors, ER+ tumors and ER‐ tumors. Protein expression and clinical data of breast cancer cohort from Tang et al. (2018) [68] was obtained from KM plotter. The patients were split into two groups based on their median BTRC protein expression. [file MOL2-16-422-s002.pdf]
